# Acid ceramidase targeting pyruvate kinase affected trypsinogen activation in acute pancreatitis

**DOI:** 10.1186/s10020-022-00538-w

**Published:** 2022-09-06

**Authors:** Juan Xiao, Wenying Zeng, Pengcheng Zhang, Yuan Zhou, Qiangqiang Fang

**Affiliations:** 1 Guangxi Key Laboratory of Molecular Medicine in Liver Injury and Repair, The Affiliated Hospital of Guilin Medical University, Guilin, 541001 Guangxi China; 2 Guangxi Health Commission Key Laboratory of Basic Research in Sphingolipid Metabolism Related Diseases, The Affiliated Hospital of Guilin Medical University, Guilin, 541001 Guangxi China

**Keywords:** Acid ceramidase, MIB1, Trypsinogen, Pyruvate kinase, Acute pancreatitis

## Abstract

**Background:**

Acute pancreatitis is the sudden inflammation of the pancreas. Severe cases of acute pancreatitis are potentially fatal and have no specific treatment available. Premature trypsinogen activation could initiate acute pancreatitis. However, the mechanism underlying premature trypsinogen activation is not fully understood.

**Methods:**

In this research, a primary pancreatic acinar cell or mouse acute pancreatitis model was constructed. The effect of acid ceramidase (ASAH1), which is responsible for sphingosine production, was investigated in trypsinogen activation in vitro and in vivo. Meanwhile, the proteins regulating ASAH1 or binding to sphingosine were also detected by co-immunoprecipitation followed by mass spectrometry.

**Results:**

The results showed that ASAH1 increased in acute pancreatitis. Increased ASAH1 promoted the activation of trypsinogen and cathepsin B. On the contrary, ASAH1 downregulation inhibited trypsinogen and cathepsin B. Meanwhile, ASAH1 regulated the activity of trypsin and cathepsin B through sphingosine. Additionally, E3 ligase Mind bomb homolog 1 (MIB1) decreased in acute pancreatitis resulting in the decreased binding between MIB1 and ASAH1. Exogenous MIB1 diminished the elevation in trypsin activity induced by acute pancreatitis inducer. ASAH1 increased owing to the inhibition of the proteasome degradation by MIB1. In acute pancreatitis, sphingosine was found to bind to pyruvate kinase. Pyruvate kinase activation could reduce trypsinogen activation and mitochondrial reactive oxygen species (ROS) production induced by sphingosine.

**Conclusions:**

In conclusion, during the process of acute pancreatitis, MIB1 downregulation led to ASAH1 upregulation, resulting in pyruvate kinase inhibition, followed by trypsinogen activation.

**Supplementary Information:**

The online version contains supplementary material available at 10.1186/s10020-022-00538-w.

## Background

Acute pancreatitis is the inflammation which occurres in pancreas. Severe cases of acute pancreatitis are potentially deadly and have no specific treatment available. Previous reports show that premature trypsinogen activation occurs in the early stage of acute pancreatitis (Mayerle et al. 2019). The mechanism underlying premature trypsinogen activation is not fully understood.

As a general rule, trypsinogen is activated by enterokinase when it is secreted into the duodenum (Saluja et al. 2007; Logsdon and Ji 2013). However, in acute pancreatitis, trypsinogen activation can be induced by cathepsin B (Saluja et al. 2019). Premature trypsinogen activation can cause pancreatic acinar injury, which may lead to acute pancreatitis.

Currently, the levels of serum sphingolipids were found to change during the process of acute pancreatitis (Kononczuk et al. 2017). Sphingolipids, which contain ceramides and sphingosine, are the components of the cell membrane, and they play important roles in cell signaling transduction (Hannun and Obeid 2018). Specifically, ceramide and sphingosine can regulate cell survival and inflammation (Maceyka and Spiegel 2014). Acid ceramidase (ASAH1) is located at the lysosome where it converts ceramide into sphingosine with the best working pH of 4.5 (Mao and Obeid 2008). It has been reported that ASAH1 could activate cathepsin B (Beckham et al. 2012). Under some conditions, exogenous sphingosine could result in cathepsin B redistribution (Talukdar et al. 2016). Although cathepsin B is closely related to premature trypsinogen activation, the roles of ASAH1 in trypsinogen activation or acute pancreatitis are not fully understood.

E3 ligase Mind bomb homolog 1 (MIB1) is a conserved RING finger E3 ubiquitin ligase regulating Notch ligands (Li et al. 2018). Meanwhile, MIB1 regulates centriolar satellite proteostasis, and MIB1-mediated degradation of Werner syndrome protein (WRN) promotes cellular senescence (Li et al. 2020). Additionally, MIB1 controls nuclear adenovirus genome release (Bauer et al. 2019). However, whether this E3 ligase regulates sphingolipid metabolism enzymes remains to be elucidated.

As the final and irreversible step in glycolysis, pyruvate kinase converts phosphoenolpyruvate and ADP into pyruvic acid and ATP. In an oxygen-deficient environment, pyruvate acid can be transformed into lactate. A previous study reported that sodium lactate injection can reduce the severity of acute pancreatitis induced by cerulein plus lipopolysaccharide (LPS) (Hoque et al. 2014). Currently, pyruvate and analogs have been found to alleviate acute pancreatitis (Yao et al. 2019). However, the relationship between pyruvate kinase and trypsinogen is still undetermined.

In this research, the roles of ASAH1 in acute pancreatitis will be elucidated in pancreatic acinar cells and mouse. At the same time, the effect of MIB1 on ASAH1 and the regulation of trypsinogen by pyruvate kinase will also be studied here.

## Methods

### Chemicals and antibodies

The following commercial antibodies were used: anti-ASAH1 (rabbit, Abcam), anti-ASAH1 (mouse, Proteintech), anti-MIB1 (rabbit, Proteintech), anti-ubiquitination (rabbit, Proteintech), anti-actin (mouse, Proteintech), anti-tubulin (rabbit, Proteintech), and anti-ceramide (mouse, Thermo). Cerulein was obtained from MedChemExpress. LPS was from Sigma-Aldrich. Sphingosine was from MedChemExpress.

### Primary pancreatic acinar cell isolation

Pancreas tissue samples were obtained from C57BL/6 mice, and collagenase was used to digest pancreatic acinar cells. After filtration, primary pancreatic acinar cells were cultured in DMEM (Gibco) supplemented with 20% FBS and used for subsequent experiments. All cultures were maintained in a 37 °C incubator with 5% CO_2_.

All animal care and experimental procedures were approved by the Ethical Committee on Animal Experiments at Guilin Medical University (approval no. GLMC201803030), and adherence to the National Institutes of Health *Guide for the Care and Use of Laboratory Animals* was ensured (NIH Publications No. 8023, revised 1978).

### Acute pancreatitis mouse construction and treatment

Male C57BL/6 mice from Hunan Slac Laboratory Animal Co. weighing 20–25 g were used. The mice were kept in a clean environment and randomly divided into four groups (n = 4). All animal care and experimental procedures complied with the *Guide for the Care and Use of Laboratory Animals* (eighth edition) and were approved by the Ethical Committee on Animal Experiments at Guilin Medical University (approval no. GLMC201803030). The mice were divided into four groups: group 1, control group; group 2, acute pancreatitis (AP) group; group 3, Ceranib-2 short treatment; and group 4, ceranib-2 long treatment. The mice from group 1 were intraperitoneally (i.p.) injected with saline seven times on an hourly basis. The mice from group 2 were i.p. injected with cerulein (50 μg/kg) seven times on an hourly basis, and at the final cerulein injection, LPS (10 mg/kg) was i.p. injected. The mice from group 3 were i.p. injected with cerulein plus LPS like in group 2, and ceranib-2 (40 mg/kg) was i.p. injected at the third injection of cerulein. The mice from group 4 were i.p. injected with cerulein plus LPS like in group 2, and ceranib-2 was i.p. injected at the first injection of cerulein.

### Trypsin activity assay

Cells or primary cells were lysed; each sample was mixed with butoxycarbonyl-Gln-Ala-Arg-7-amido-4-methylcoumarin hydrochloride (Sigma) in trypsin reaction buffer (10 mM of Tris and 20 mM of CaCl_2_, pH 7.4) and then incubated for 30 min at 37 °C. The fluorescence intensity from the trypsin substrate was measured at 450 nm in a fluorescence microplate reader under excitation at 380 nm (Xiao et al. 2020). Trypsin activity was also detected by fluorescence microscopy using the trypsin substrate rhodamine 110, bis-(CBZ-l-isoleucyl-l-prolyl-l-arginine amide), dihydrochloride (BZiPAR) (Invitrogen) (Grasso et al. 2011).

### Cathepsin B activity assay

Cathepsin B activity was measured according to the Enzymatic Assay of Cathepsin B protocol provided by Sigma Inc. In brief, cell or tissue lysate was incubated with cathepsin B substrate Nα-CBZ-Arg-Arg-7-amido-4-methylcoumarin at 37 °C, and the fluorescence intensity was determined at 440 nm in a fluorescence microplate reader under excitation at 348 nm (Xiao et al. 2020).

### Western blotting

The lysates from cells or animal pancreas were resolved by SDS–polyacrylamide gel electrophoresis and transferred to PVDF membranes (Millipore). After being probed with indicated primary antibodies and then anti-rabbit or anti-mouse IgG secondary antibodies (PTG), the reactions were visualized by chemiluminescence reagents.

### Immunostaining

Cells were fixed with 4% paraformaldehyde and then penetrated with 0.1% Triton X-100. The cells were blocked with 5% goat serum. Anti-ceramide antibody was added to the cells at 37 °C for 1 h. Goat polyclonal Secondary Antibody to Mouse IgG-H&L (Alexa Fluor® 647) was added to the cells at room temperature for 1 h. Ceramide signaling was observed by fluorescence microscopy.

### Co-immunoprecipitation of ASAH1

Cells were lysed with immunoprecipitation lysis buffer (Solarbio) containing protease and Phosphatase Inhibitor Cocktail (MedChemExpress). The lysate was centrifugated (12,000 rpm, 10 min). For the ASAH1 binding assay, the supernatant was removed and then incubated overnight with an anti-ASAH1 antibody (mouse) at 4 °C. Protein A/G Magnetic Beads (MedChemExpress) were added according to the instructions. Finally, beads were separated and washed. Proteins binding to the ASAH1 on beads were analyzed by Western blotting.

### Biotin-sphingosine binding assay

Cells were treated with saline or cerulean plus LPS and then were lysed. Cell lysate with indicated treatments was incubated with biotin or biotin-sphingosine (J&K Scientific Ltd.). Protein binding to biotin or biotin-sphingosine enriched in the streptavidin magnetic beads (MedChemExpress) was analyzed by mass spectrometry.

### Statistical analysis

Error bars for enzyme activity were presented as the standard deviation of triplicate samples. One-way Analysis of Variance was used as statistical analysis by using SPSS. *P < 0.05, **P < 0.01, and ***P < 0.001.

## Results

### The protein level of ASAH1 increased in acute pancreatitis

**Fig. 1:**
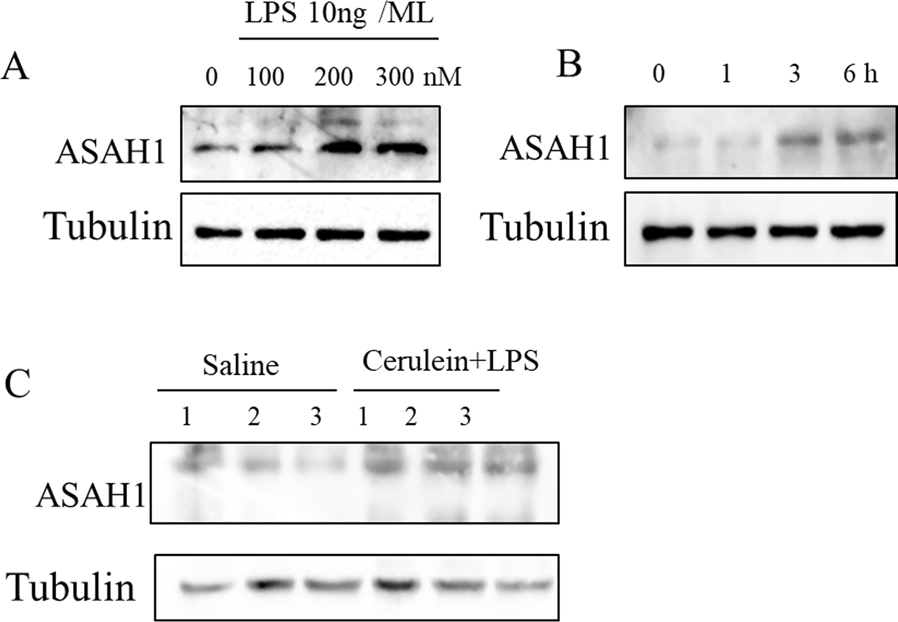
Acid ceramidase (ASAH1) was increased in acute pancreatitis. **A**, **B** primary pancreatic acinar cells were treated with different concentrations of cerulein plus LPS (10 ng/mL) for 6 h (**A**) or treated with 200 nM of cerulein plus LPS (10 ng/mL) for indicated durations (**B**). **C** Mice injected with saline or cerulein plus LPS were sacrificed, and their pancreas samples were collected and lysed. Tissue lysate was examined by Western blotting with indicated antibodies (**C**)

To investigate the roles of ASAH1 in acute pancreatitis, we first detected the protein level of ASAH1 in pancreatic cells under the cerulein plus LPS treatment. The results showed that the cerulein plus LPS treatment induced the protein expression of ASAH1 in a dose- and time-dependent manner (Fig. 1A, B). As the results show, the protein level of ASAH1 increased by 100 nM or at a higher concentration of the cerulein plus LPS treatment (Fig. 1A). Meanwhile, 3 h or longer treatment of cerulein plus LPS induced ASAH1 protein expression (Fig. 1B). We used the 200 nM cerulein treatment for 6 h for the next analysis. Additionally, we induced acute pancreatitis in mice by i.p. injection of cerulein plus LPS. Consistent with the results in acinar cells, ASAH1 in the pancreas of mice with acute pancreatitis increased (Fig. 1C).

### ASAH1 promoted trypsinogen and cathepsin B activation

**Fig. 2:**
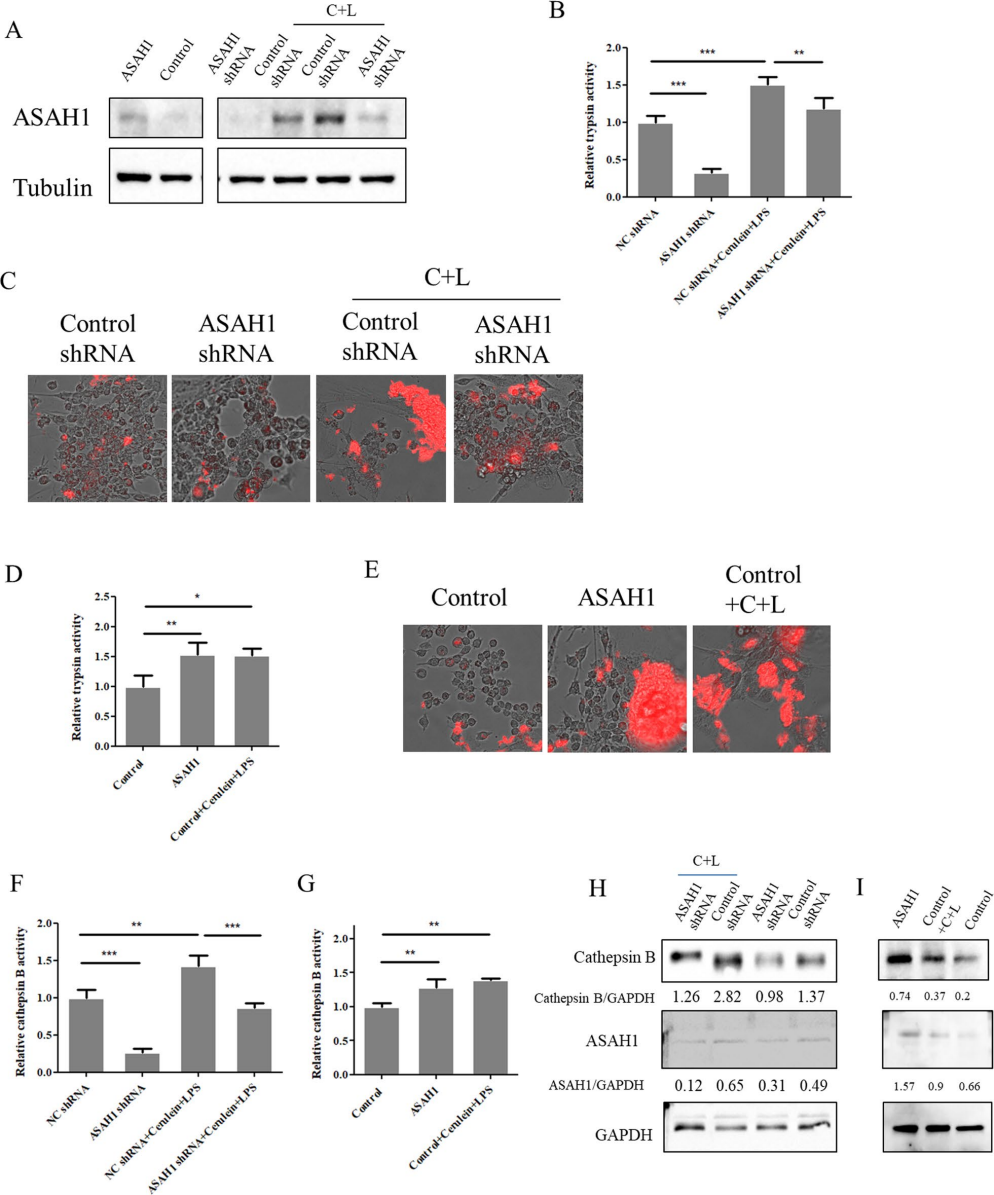
Acid ceramidase (ASAH1) positively regulated the activity of trypsin and cathepsin B. Lentivirus particles containing nontargeting shRNA or shRNA targeting ASAH1 (**A**, **B**, **E**, **G**) and control vector or plasmid encoding ASAH1 (**A**, **C**, **D**, **F**, **H, I**) were added to primary pancreatic acinar cells. Additionally, the cells were treated with or without 200 nM of cerulein plus 10 ng/mL of LPS (C + L) for 6 h. Cells were lysed, a proportion of lysate was examined by trypsin and cathepsin B activity assay using a microplate reader (**A**, **C**, **E**, **F**), and the other proportion was subjected to Western blotting analysis with indicated antibodies (**G**, **H, I**). Cells were incubated with trypsin rhodamine-conjugated substrate and were visualized with a fluorescence microscope (**B**, **D**)

ASAH1 could be increased in acute pancreatitis, indicating that this enzyme may influence the initiation of acute pancreatitis. To test this hypothesis, we knocked down ASAH1 with specific shRNA. ASAH1 knockdown resulted in trypsin activity inhibition as the invisible and visible fluorescence intensity of the trypsin substrate decreased (Fig. 2A–C). Meanwhile, ASAH1 knockdown also reduced the trypsin activity induced by the cerulein plus LPS treatment (Fig. 2A–C). On the contrary, ASAH1 overexpression promoted trypsinogen activation (Fig. 2A, D, E). Cathepsin B is well known to stimulate trypsinogen in acute pancreatitis. Therefore, cathepsin B activity was analyzed next. Measurements using a spectrophotometer showed that cathepsin B was inhibited under ASAH1 knockdown and promoted under ASAH1 overexpression (Fig. 2F, G). Consistently, Western blotting analysis results suggested that the level of active cathepsin B decreased when ASAH1 was knocked down with or without the cerulein plus LPS treatment, whereas active cathepsin B increased when ASAH1 was overexpressed (Fig. 2H, I).

### ASAH1 inhibitor ameliorated the pathology of acute pancreatitis

**Fig. 3:**
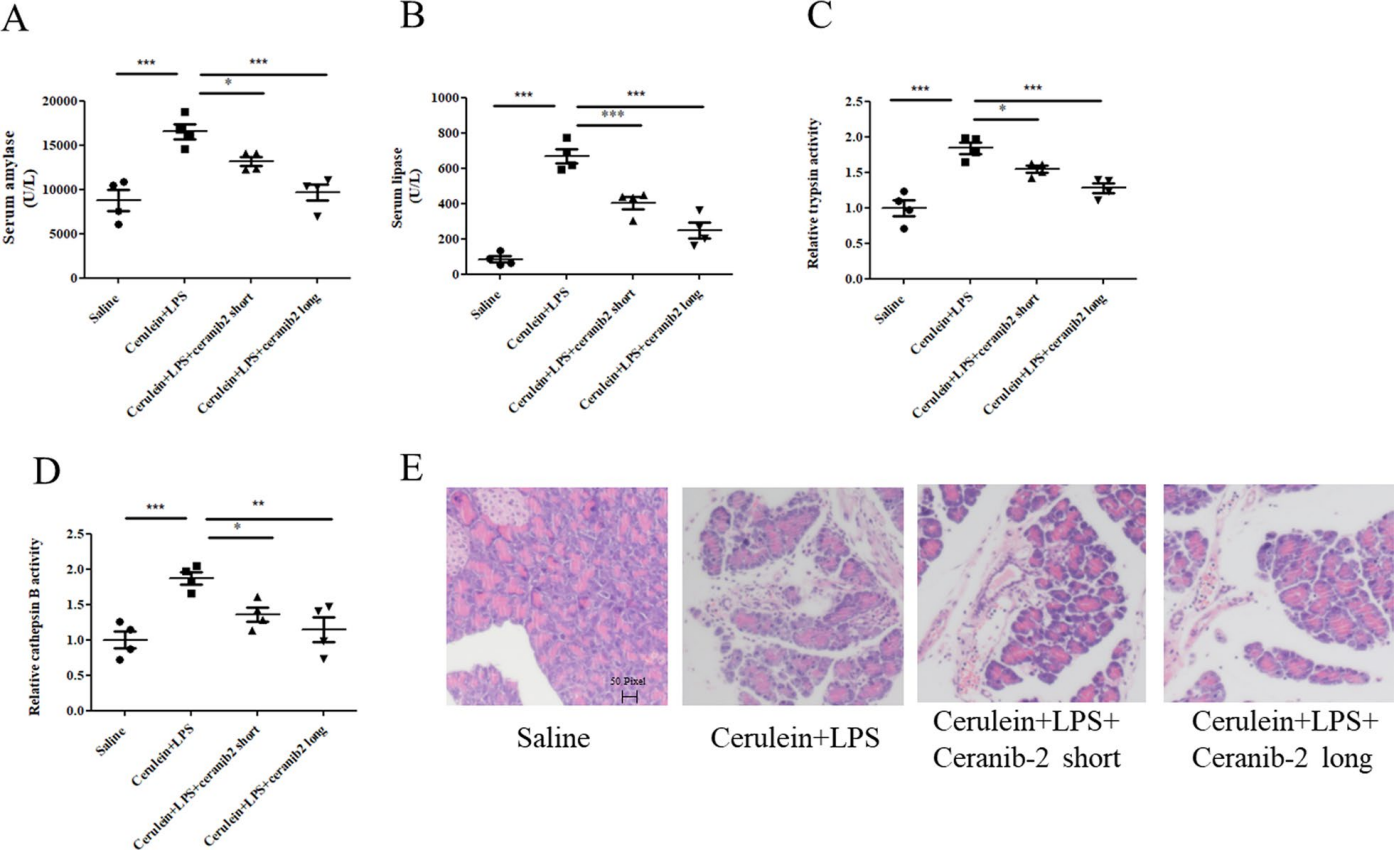
Acid ceramidase (ASAH1) inhibitors ameliorated the pathology of acute pancreatitis in vivo. Mice were treated as indicated, and serum and pancreas samples were collected. Lipase (**A**) and amylase (**B**) in serum were measured. A proportion of their pancreas samples was subjected to trypsin (**C**) and cathepsin B (**D**) analyses and HE staining (**E**)

Cellular experiments showed that ASAH1 had the potential to be a risk factor for acute pancreatitis and that ASAH1 inhibition may be useful for acute pancreatitis treatment. Therefore, we next investigated the therapeutic potential of ASAH1 inhibitor, Ceranib-2, in acute pancreatitis. We i.p. injected Ceranib-2 for short or long term. As we expected, this compound could reduce the levels of serum amylase and lipase increased by cerulein plus LPS treatment (Fig. 3A, B). Pancreatic trypsin activity and cathepsin B were also inhibited by Ceranib-2 (Fig. 3C, D). HE staining images showed that edema in the pancreas in acute pancreatitis seemed to be reduced by Ceranib-2 (Fig. 3E). Note that long-term treatment of Ceranib-2 worked better in acute pancreatitis mice.

### ASAH1 regulated trypsinogen and cathepsin B activation dependent on sphingosine

**Fig. 4:**
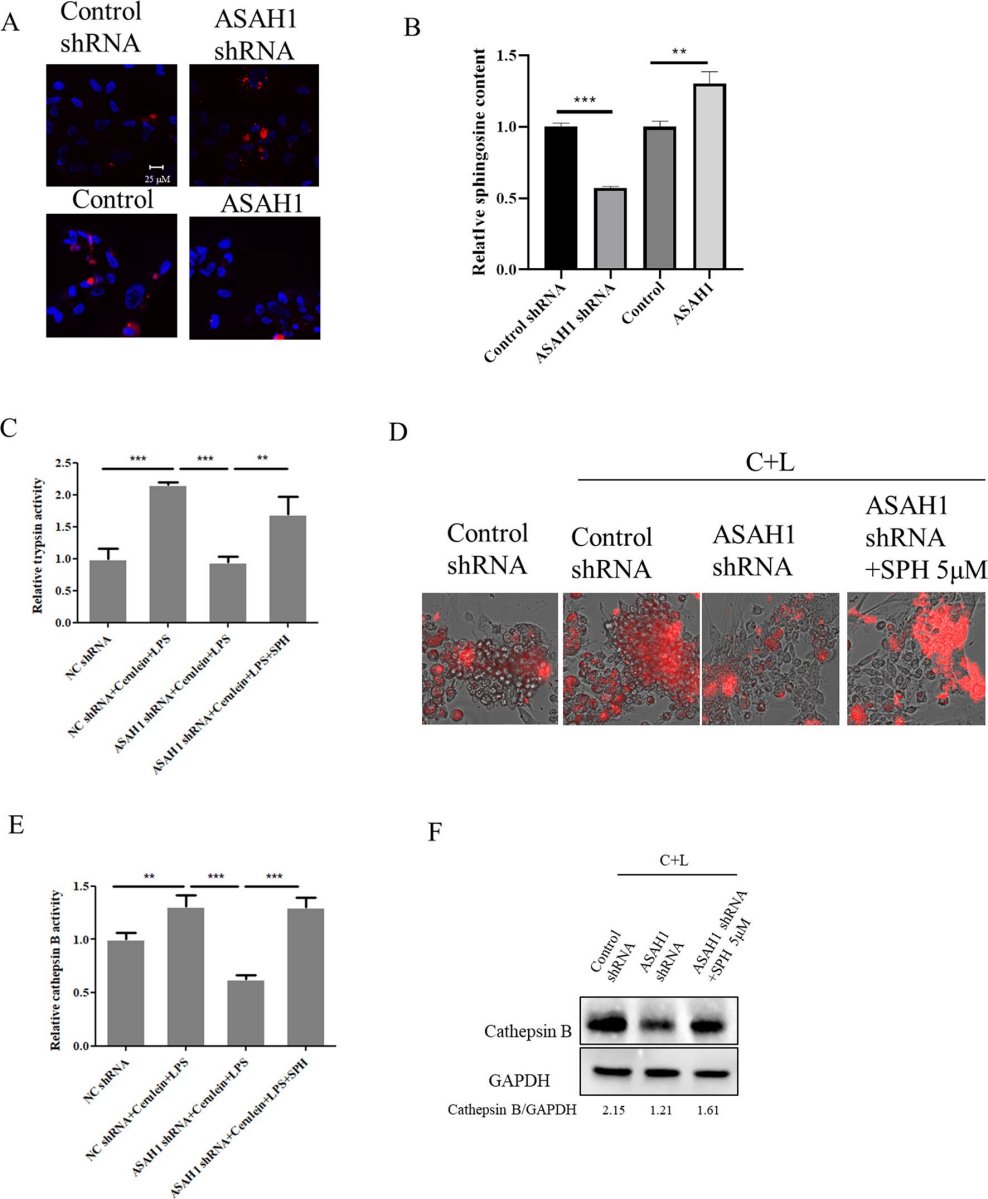
Acid ceramidase (ASAH1) regulated the activity of trypsin and cathepsin B through sphingosine (SPH). Lentivirus particles containing nontargeting shRNA/Control vector or shRNA targeting ASAH1/ASAH1 expressing plasmid were added to primary pancreatic acinar cells. Additionally, the cells were treated as indicated. Cells were fixed, and immunostaining was performed with an anti-ceramide antibody (**A**). Trypsin activity was detected by a spectrophotometer (**B**) or fluorescence microscope (**C**). Cathepsin B activity was detected by a spectrophotometer (**D**) or Western blotting (**E**, **F**)

ASAH1 is the enzyme that converts ceramides into sphingosine. Therefore, we detected ceramides by immunostaining using the anti-ceramidase antibody. We found that the intensity of ceramide signaling was induced in the case of ASAH1 knockdown (Fig. 4A). ASAH1 overexpression led to the downregulation of ceramides (Fig. 4A). Meanwhile, we found sphingosine to increase and decrease in pancreatic acinar cells when ASAH1 was overexpressed and reduced, respectively (Fig. 4B). These results indicated that ceramide or sphingosine may regulate the activity of trypsin and cathepsin B. We added sphingosine to pancreatic acinar cells under ASAH1 knockdown. We found that sphingosine reverses the effect of downregulated ASAH1 on trypsin and cathepsin B (Fig. 4C–F).

### Decreased MIB1 induced ASAH1 in acute pancreatitis

**Fig. 5:**
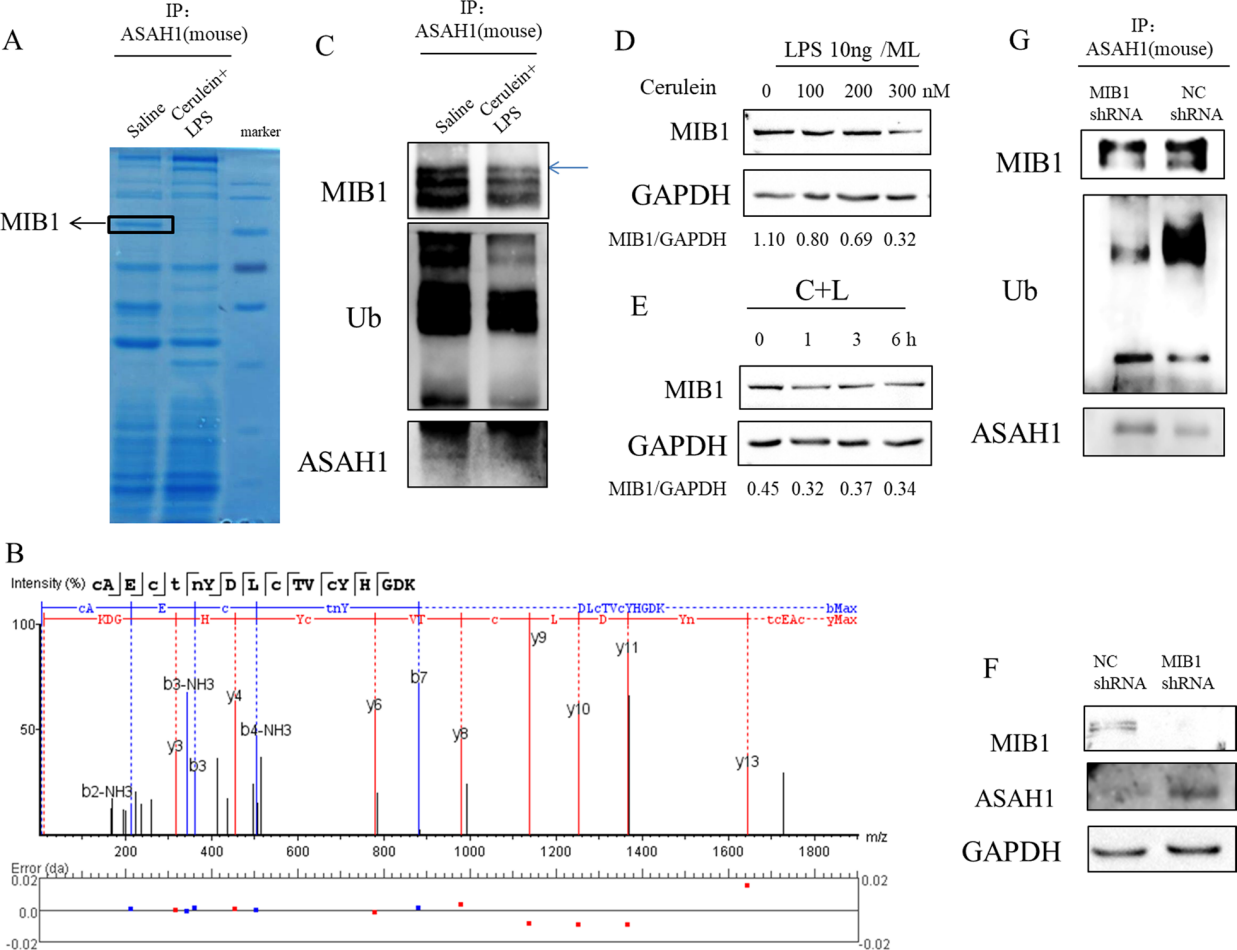
E3 ligase Mind bomb homolog 1 (MIB1) promoted proteasomal degradation of acid ceramidase (ASAH1). **A**–**C** Primary pancreatic acinar cells were treated with saline or 200 nM of cerulein plus 10 ng/mL of LPS for 6 h. Cells were lysed, and the lysate was subjected to immunoprecipitation with an anti-ASAH1 antibody. The proteins binding to ASAH1 were denatured and separated by SDS–polyacrylamide gel electrophoresis (SDS–PAGE) gel. **A**, **B** The gel was stained with Coomassie brilliant blue (**A**), and the protein band with square was detected by mass spectrometry (n = 1) (**B**). The proteins on the gel were transferred to the PVDF membrane and visualized by ECL substrate (**C**). **D**, **E** Primary pancreatic acinar cells were treated with different concentrations of cerulein plus LPS (10 ng/mL) for 6 h (**D**) or treated with 200 nM of cerulein plus LPS (10 ng/mL) for indicated durations (**E**); cells were lysed, and cell lysate was examined by Western blotting with indicated antibodies. **F**, **G** Lentivirus particles containing nontargeting shRNA or shRNA targeting MIB1 were added to primary pancreatic acinar cells. Cells were lysed, a proportion of cell lysate was analyzed by Western blotting (**F**), and the other proportion was subjected to immunoprecipitation (**G**)

The aforementioned results showed that the protein level of ASAH1 increased in acute pancreatitis. To understand the cause of ASAH1 upregulation, we analyzed the protein binding to ASAH1 by co-immunoprecipitation. We separated the binding proteins in SDS–polyacrylamide gel electrophoresis gel and stained them with Coomassie brilliant blue. We found that the band was present in the saline-treated sample but absent in the cerulein plus LPS-treated sample (Fig. 5A). We sliced the gel containing the band marked with a black square frame and examined it by mass spectrometry. Specifically, we identified E3 ligase MIB1 (Fig. 5B). Next, we investigated the relationship between ASAH1 and MIB1.

We performed a co-immunoprecipitation experiment followed by Western blotting with indicated antibodies to validate the mass spectrometry result. Compared to the saline treatment, the cerulein plus LPS treatment reduced the binding between ASAH1 and MIB1 (Fig. 5C). The extent of ASAH1 ubiquitination was also decreased in acinar cells under the cerulein plus LPS treatment (Fig. 5C). We analyzed the protein expression of MIB1 in pancreatic cells treated with cerulein plus LPS at different concentrations or at different times. Contrary to the changes in ASAH1, the cerulein plus LPS treatment inhibited MIB1 protein expression in a dose- and time-dependent manner (Fig. 5D, E). Meanwhile, MIB1 knockdown led to ASAH1 upregulation and ASAH1 ubiquitination downregulation (Fig. 5F, G). Taken together, E3 ligase downregulation resulted in the inhibition of the proteasome degradation of ASAH1 in acute pancreatitis.

### ASAH1-mediated trypsinogen activation could be regulated by MIB1

**Fig. 6:**
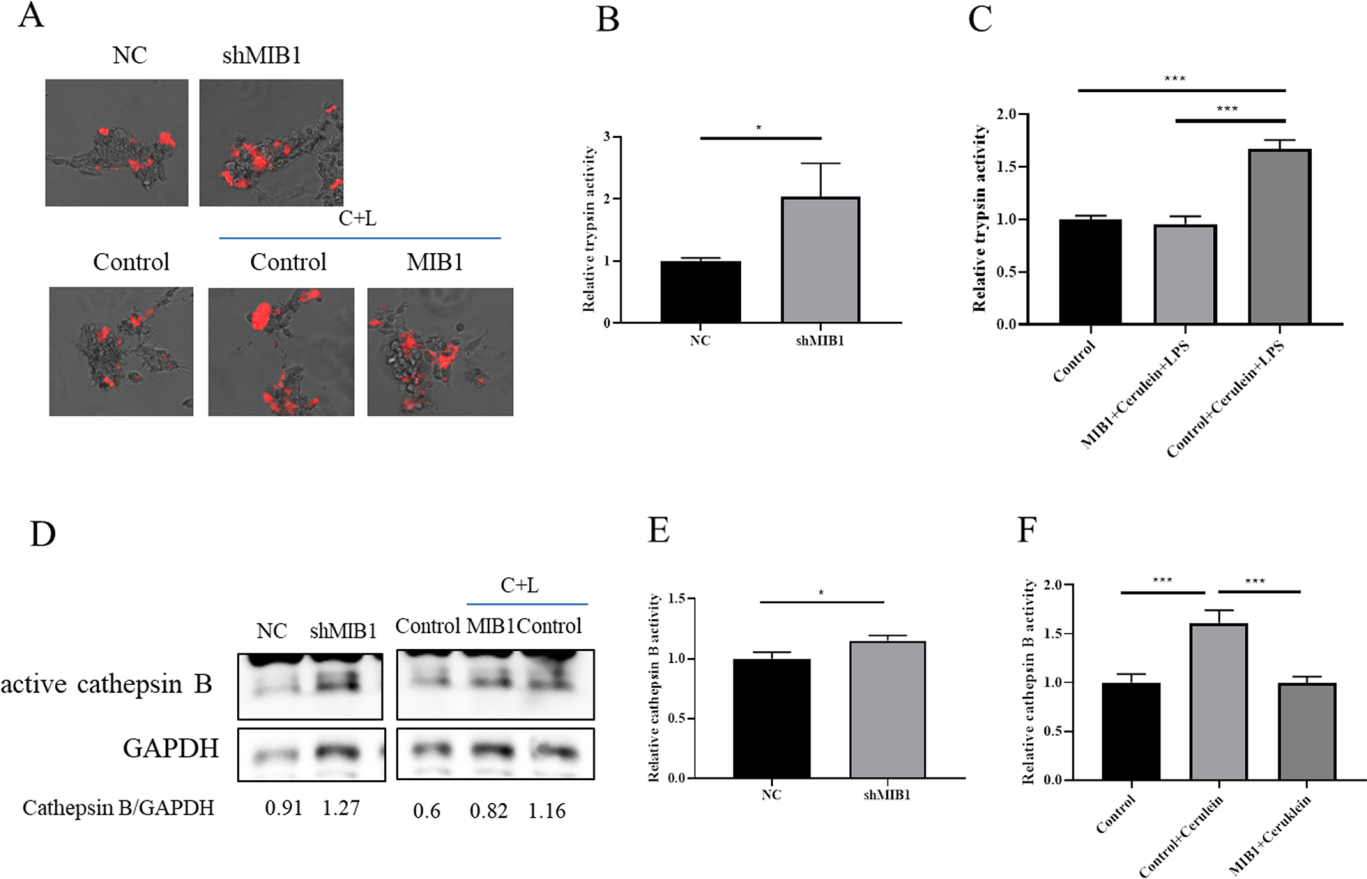
Acid ceramidase (ASAH1)-mediated trypsinogen activation could be regulated by Mind bomb homolog 1 (MIB1). Lentivirus particles containing indicated plasmids were added to the cells. Trypsin activity was detected by a fluorescence microscope (**A**) or spectrophotometer (**B**, **C**). Cathepsin B activity was detected by Western blotting (**D**) or a spectrophotometer (**E**, **F**)

Because MIB1 could regulate ASAH1, we presumed that MIB1 was involved in the process of trypsinogen activation. We found that MIB1 knockdown increased the activity of trypsin and cathepsin B (Fig. 6A, B, D, E), whereas exogenous MIB1 inhibited trypsinogen and cathepsin B activation induced by the cerulein plus LPS treatment (Fig. 6A, C, D, F).

### Sphingosine binding to pyruvate kinase regulated trypsinogen activation

**Fig. 7:**
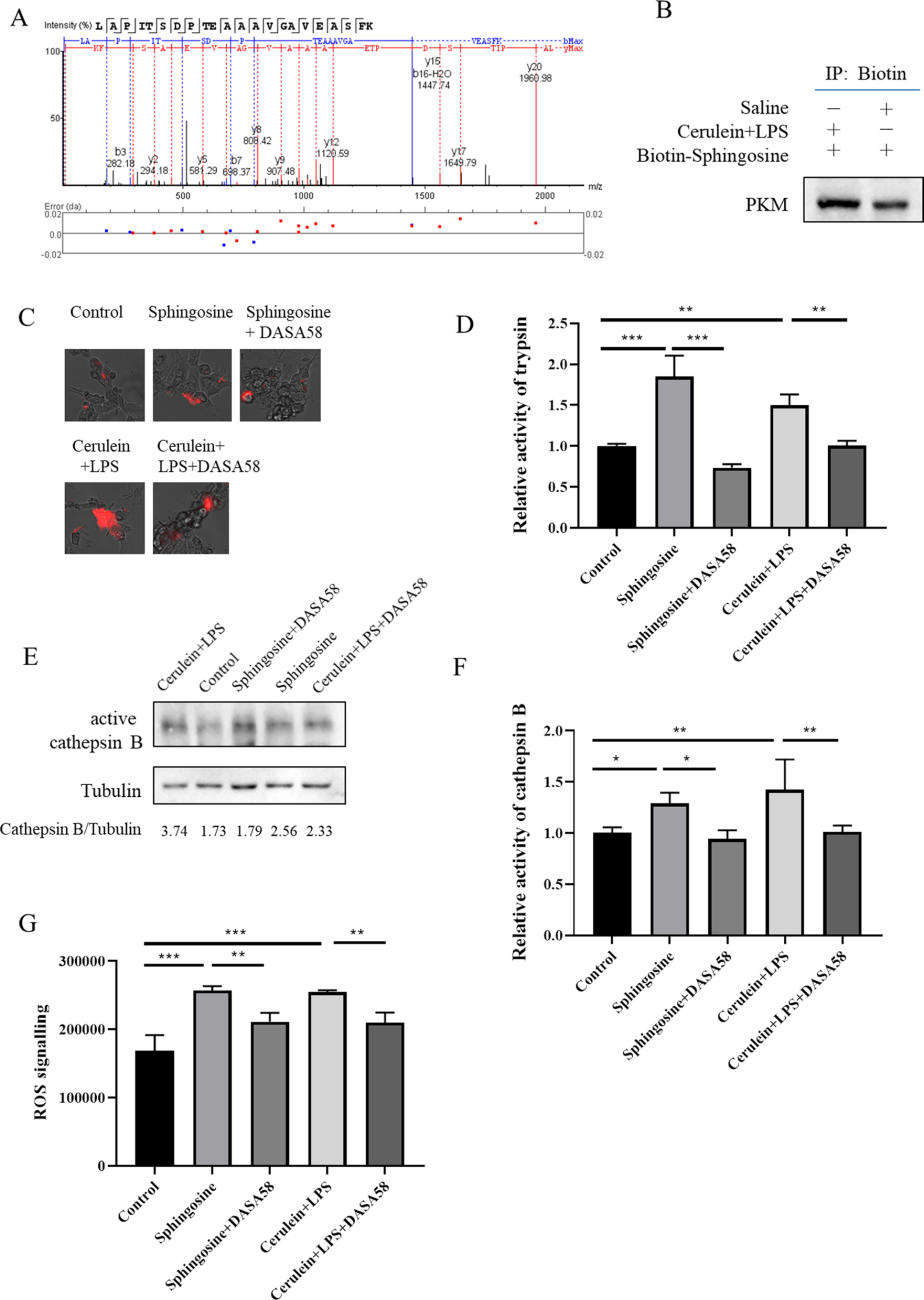
SPH binding to pyruvate kinase regulated trypsinogen activation. Primary pancreatic acinar cells were treated with saline or 200 nM of cerulein plus 10 ng/mL of LPS for 6 h. Cells were lysed, and the lysate was incubated with biotin-conjugated sphingosine. Proteins binding to the sphingosine were enriched in streptavidin magnetic beads. The potential target identified by mass spectrometry is shown (**A**) and validated by Western blotting (**B**). **C**–**G** Primary cells were treated with solvent, 5 μM of sphingosine, 10 μM of DASA58 plus sphingosine, and 200 nM of cerulein plus 10 ng/mL of LPS and DASA58 with LPS and sphingosine for 6 h. Cells were lysed for trypsin (**C**, **D**) and cathepsin B (**E**, **F**) assay, or were stained with mitochondrial ROS-specific dye, and detected by FACS (**G**)

The aforementioned results showed that ASAH1 regulated the activity of trypsin through sphingosine. We further investigated the mechanism underlying trypsinogen activation induced by ASAH1 in connection with the relationship between sphingosine and trypsin. We applied biotin–streptavidin system. We analyzed the proteins binding to the biotin-conjugated sphingosine under the cerulein plus LPS treatment by mass spectrometry. The amount of each protein binding to biotin/biotin-sphingosine under saline or cerulein plus LPS treatment is shown in Additional file 2: Table S1. Comparing the ratio of biotin-sphingosine mass signaling area and biotin area to the cerulein plus LPS treatment with the ratio of biotin-sphingosine area and biotin area to saline, we surmised that pyruvate kinase is the target of sphingosine (Additional file 2: Table S1, Fig. 7A). Western blotting analysis validated the binding between pyruvate kinase and sphingosine (Fig. 7B).

We used AutoDock Vina software (1.1.2) to predict the interaction pattern between sphingosine and pyruvate kinase. The results suggested that amino acid residues Gln329, Gly298, and Asp178 on pyruvate kinase form a hydrogen bond with sphingosine and that a hydrophobic effect exists among the amino acid residues Gly295, Val306, Gln310, Ile299, Phe307, Asp347, Ala303, and Arg294 on pyruvate kinase and sphingosine (Additional file 1: Fig. S1). Therefore, sphingosine had the potential to block pyruvate kinase.

To illustrate the role of pyruvate kinase in trypsinogen activation, we used the activator of the kinase DASA58. We found that DASA58 decreased the activity of trypsin and cathepsin B under sphingosine or cerulein plus LPS treatment (Fig. 7C–F). Meanwhile, DASA58 could also reduce the level of mitochondrial ROS induced by sphingosine or cerulein plus LPS treatment (Fig. 7G).

## Discussion

In our research, ASAH1 was found to be increased in acute pancreatitis. Increased ASAH1 promoted trypsinogen and cathepsin B activation. Opposite results were observed when ASAH1 was inhibited. Meanwhile, ASAH1 regulated the activity of trypsin and cathepsin B through sphingosine. Additionally, E3 ligase MIB1 decreased in acute pancreatitis, resulting in the inhibition of the proteasome degradation of ASAH1. Exogenous MIB1 could reverse trypsinogen activation induced by the cerulein plus LPS treatment. Finally, sphingosine was found to target pyruvate kinase, which may influence the production of mitochondrial ROS.

Currently, ASAH1 has been reported to be associated with drug resistance and is a therapeutic target for cancer (Tan et al. 2019; Vijayan et al. 2019). Here we found that ASAH1 was involved in acute pancreatitis development, positively regulating trypsinogen, whereas premature trypsin could induce cell death eventually. It seemed that the roles of ASAH1 in cell death in inflammation may be different from those in cancer. Specifically, ASAH1 transformed ceramide into sphingosine, and the latter could be converted into sphingosine-1-phosphate. Both ceramide and sphingosine can induce cell death, but sphingosine-1-phosphate can promote cell survival. Thus ASAH1 may work with other proteins in cancer, which may be different in acute pancreatitis.

In this research, cathepsin B activity could be affected by ASAH1, and this regulation was dependent on sphingosine. Sphingosine has been reported to influence the distribution of cathepsin B (Jakkampudi et al. 2016). Therefore, ASAH1 may regulate the location of cathepsin B in acute pancreatitis. Note that the activity of cathepsin B could be affected by the pH in lysosome (Waterford et al. 2005; Bhoomagoud et al. 2009). Because pH in the lysosome was regulated by H+-ATPase and sphingosine was present on the lysosome membrane, sphingosine or ASAH1 may affect the H+-ATPase to some extent.

Currently, exogenous ASAH1 was found to reduce inflammation in hepatic ischemia/reperfusion injury, which was presumably associated with the production of sphingosine-1-phosphate caused by sphingosine kinase activity upregulation (Jiang et al. 2021). In our research, ASAH1 promoted inflammation, which may be related to sphingosine-1-phosphate hydrolase activation.

A previous report showed that increased cellular calcium could induce ASAH1 expression (Sun et al. 2008). In our research, ASAH1 upregulation was caused by the inhibition of its degradation. The interaction between E3 ligase MIB1 and ASAH1 was inhibited. Meanwhile, the ubiquitination of ASAH1 was reduced. These results suggested that MIB1 may be the specific ligase for ASAH1, which needs to be investigated further. It is well known that intracellular calcium overload occurs in the early stage of acute pancreatitis. Therefore, MIB1 downregulation in acute pancreatitis may presumably be related to calcium overload.

ASAH1 knockdown in our study could inhibit trypsinogen activation at the same time. ASAH1 may be the potential therapeutic target for acute pancreatitis. In severe cases of acute pancreatitis, necrosis presents in the pancreas. However, the effect of ASAH1 in necrosis was not investigated here.

Pyruvate kinase is overactive in tumors, contributing to the Warburg effect and promoting cell survival in an oxygen-deficient environment (Rajala 2020). Trypsin-induced cell death occurs in the late stage of acute pancreatitis when hypoxia is present (Karne and Gorelick 1999). However, sphingosine targeting pyruvate kinase activates trypsinogen in the early stage of acute pancreatitis. Therefore, pyruvate kinase may work in both the early and late stages of acute pancreatitis.

During the process of acute pancreatitis, mitochondrial dysfunction leads to ATP reduction (Gerasimenko et al. 2018). In our study, pyruvate kinase was inhibited by sphingosine in acute pancreatitis, whereas pyruvate kinase was responsible for ATP production in glycolysis. ATP decrement in acute pancreatitis may be partly caused by pyruvate kinase inhibition. Meanwhile, glycolysis may be involved in the pathology of acute pancreatitis.

ROS signaling pathway is upregulated in acute pancreatitis. Additionally, pyruvate could work as an antioxidant (Mallet et al. 2018). Consistently, our study indicated that pyruvate kinase regulated mitochondrial ROS content. Taken together, pyruvate kinase may inhibit trypsinogen by reducing ROS. Note that pyruvate kinase translocated to the mitochondria under oxidation stress, which may be present in acute pancreatitis (Liang et al. 2017).

## Conclusions

**Fig. 8:**
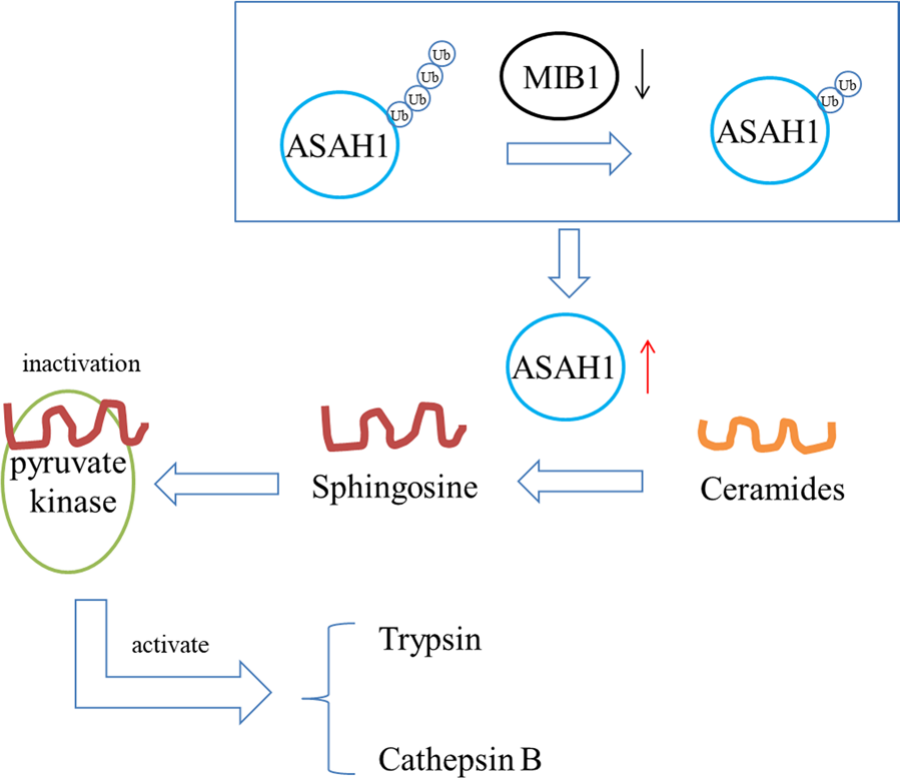
Working model of Mind bomb homolog 1 (MIB1)/acid ceramidase (ASAH1)/sphingosine/pyruvate kinase axis regulating trypsinogen activation in acute pancreatitis

In conclusion, ASAH1 could promote trypsinogen activation. ASAH1 could be treated as a therapeutic target for acute pancreatitis. MIB1/ASAH1/sphingosine/pyruvate kinase axis regulated trypsinogen activation in acute pancreatitis (Fig. 8).

## Supplementary Information

**Additional file 1: Figure S1.** Sphingosine and private kinase binding pattern.

**Additional file 2: Table S1.** Proteins binding to biotin or biotinsphingosine under saline or cerulein plus LPS.

## Data Availability

Not applicable.

## Abbreviations

AP: Acute pancreatitis
ASAH1: Acid ceramidase
MIB1: Mind bomb homolog 1
I.P.: Intraperitoneally
BZiPAR: Rhodamine 110 bis-(CBZ-l-isoleucyl-l-prolyl-l-arginine amide) dihydrochloride
PAGE: SDS–polyacrylamide gel electrophoresis
LPS: Lipopolysaccharide
