# Supplementary material for: Acid ceramidase targeting pyruvate kinase affected trypsinogen activation in acute pancreatitis
Source: Mol Med. 2022 Sep 6;28:106. doi: 10.1186/s10020-022-00538-w (PMC9450262; doi:10.1186/s10020-022-00538-w)
Supplement: Supplementary file 1 — Additional file 1: Figure S1. Sphingosine and private kinase binding pattern. [file 10020_2022_538_MOESM1_ESM.pdf]

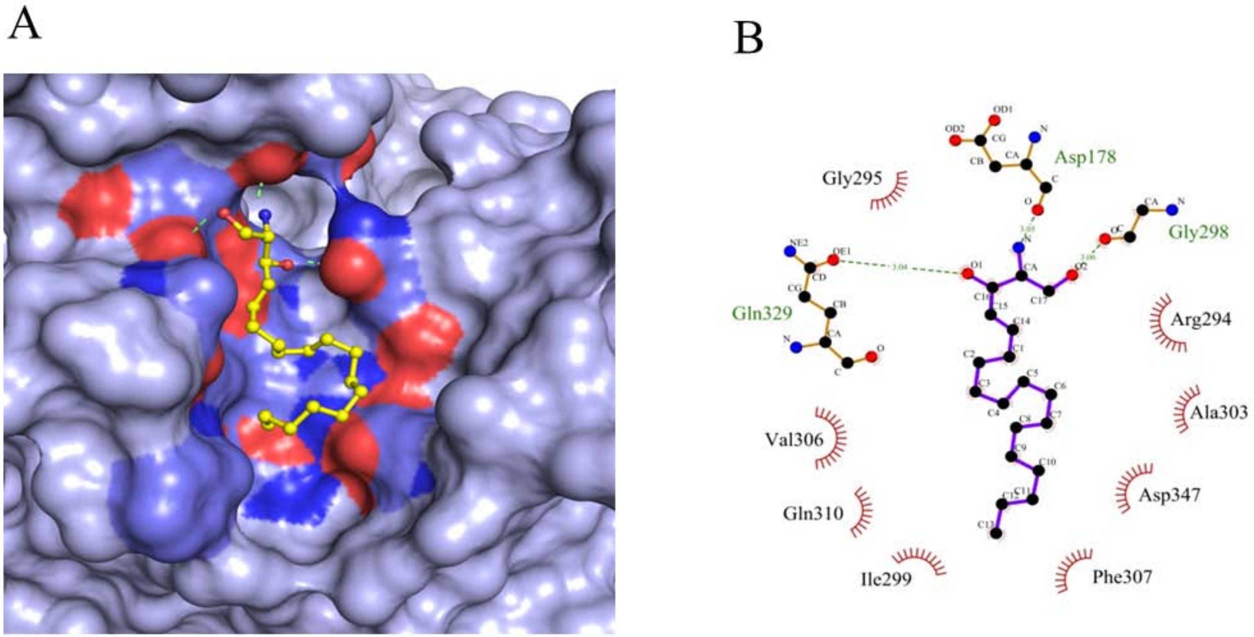

Figure S1. Sphingosine and private kinase binding pattern. AutoDock Vina software (1.1.2) was used to predict the interaction pattern between sphingosine and pyruvate kinase. The 3D binding image (A) and the specific binding sites (B) were shown.
