# Supplementary material for: Acid ceramidase targeting pyruvate kinase affected trypsinogen activation in acute pancreatitis
Source: Mol Med. 2022 Sep 6;28:106. doi: 10.1186/s10020-022-00538-w (PMC9450262; doi:10.1186/s10020-022-00538-w)
Supplement: Supplementary file 2 — Additional file 2: Table S1. Proteins binding to biotin or biotinsphingosine under saline or cerulein plus LPS. [file 10020_2022_538_MOESM2_ESM.pdf]

Table S1. Proteins binding to biotin or biotinsphingosine under saline or cerulein plus LPS.

Accession	Area Cerulein+LPS-Biotin	Area Cerulein+LPS-Biotinsphingosine	Area Saline-Biotin	Area Saline-Biotinsphingosine	Avg. Mass	Description
Q8VDD5	8189200000	9056200000	1167200000	9834500000	226370	Myosin-9 OS=Mus musculus OX=10090
P35441	1028900000	1201200000	626660000	937620000	129647	Thrombospondin-1 OS=Mus musculus
Q9JKF1	504500000	594570000	322080000	533510000	188741	Ras GTPase-activating-like protein IQGAP1 OS=Mus musculus OX=10090 GN=Iqgap1 PE=1 SV=2
Q6URW6	252220000	288340000	24876000	365140000	228584	Myosin-14 OS=Mus musculus OX=10090
Q61879	50161000	62722000	7244100	97050000	228994	Myosin-10 OS=Mus musculus OX=10090
P60710	201180000	210650000	39889000	190190000	41737	Actin cytoplasmic 1 OS=Mus musculus OX=10090 GN=Actb
P63260	98474000	110750000	18406000	128930000	41793	Actin cytoplasmic 2 OS=Mus musculus OX=10090 GN=Actg1
Q7TPV4	184780000	236110000	129510000	221100000	152036	Myb-binding protein 1A OS=Mus musculus OX=10090
P29268	732620000	605590000	367920000	572060000	37824	CCN family member 2 OS=Mus musculus OX=10090 GN=Ccn2
P26041	264430000	251950000	218560000	249660000	67767	Moesin OS=Mus musculus OX=10090
P05784	487810000	496300000	55139000	306450000	47538	Keratin type I cytoskeletal 18 OS=Mus musculus OX=10090
P11679	230670000	235630000	41431000	156030000	54565	Keratin type II cytoskeletal 8 OS=Mus musculus OX=10090
Q9Z0N1	599720000	576520000	376720000	533640000	51066	Eukaryotic translation initiation factor 2 subunit 3 X-linked OS=Mus musculus OX=10090
P99024	114510000	137800000	77507000	139910000	49671	Tubulin beta-5 chain OS=Mus musculus OX=10090 GN=Tubb5
P68372	52844000	57452000	32822000	50255000	49831	Tubulin beta-4B chain OS=Mus musculus OX=10090 GN=Tubb4b
Q61656	172950000	162040000	102350000	158180000	69290	Probable ATP-dependent RNA helicase DDX5 OS=Mus musculus
P26040	126610000	106190000	84619000	106130000	69407	Ezrin OS=Mus musculus OX=10090 GN=Ezr
P68369	1751700	2821100	1343000	3687400	50136	Tubulin alpha-1A chain OS=Mus musculus OX=10090 GN=Tuba1a
P62737	835290	904660		429510	42009	Actin aortic smooth muscle OS=Mus musculus OX=10090
O08638	52703000	63989000	791150	30640000	227026	Myosin-11 OS=Mus musculus OX=10090
P68033	0				42019	Actin alpha cardiac muscle 1 OS=Mus musculus OX=10090

P62082	895010000	965020000	612420000	1337400000	22127	40S ribosomal protein S7 OS=Mus musculus OX=10090 GN=Rps7
P68373	4247900	5589900	4298900	5856000	49909	Tubulin alpha-1C chain OS=Mus musculus OX=10090 GN=Tuba1c
P97351	454740000	424430000	360780000	472180000	29885	40S ribosomal protein S3a OS=Mus musculus OX=10090 GN=Rps3a
P68134	258000	767790			42051	Actin alpha skeletal muscle OS=Mus musculus OX=10090
Q91ZA3	155130000	132380000	96226000	171790000	79922	Propionyl-CoA carboxylase alpha chain mitochondrial OS=Mus
Q05D44	135850000	127500000	76650000	150730000	137616	Eukaryotic translation initiation factor 5B OS=Mus musculus
Q7TMM9	1770600	1994000	1053100	1930200	49907	Tubulin beta-2A chain OS=Mus musculus OX=10090 GN=Tubb2a
Q9CWF2	1966500	3391500	1670700	2634400	49953	Tubulin beta-2B chain OS=Mus musculus OX=10090 GN=Tubb2b
P26043	50136000	54134000	37679000	46970000	68543	Radixin OS=Mus musculus OX=10090
P63268	724350	10382000	1813400	349970	41877	Actin gamma-enteric smooth muscle OS=Mus musculus OX=10090
Q8BTM8	56322000	87735000	17097000	55033000	281219	Filamin-A OS=Mus musculus OX=10090
P68368	23905000	22562000	12859000	23128000	49924	Tubulin alpha-4A chain OS=Mus musculus OX=10090 GN=Tuba4a
Q6ZWX6	246960000	244100000	153630000	242910000	36108	Eukaryotic translation initiation factor 2 subunit 1 OS=Mus musculus
P18406	258530000	225400000	164960000	251700000	41709	CCN family member 1 OS=Mus musculus OX=10090 GN=Ccn1
P62702	321660000	315990000	218970000	373950000	29598	40S ribosomal protein S4 X isoform OS=Mus musculus OX=10090
Q99MN9	202990000	208060000	80209000	213130000	58409	Propionyl-CoA carboxylase beta chain mitochondrial OS=Mus
Q80X90	27409000	52057000	3686700	24509000	277822	Filamin-B OS=Mus musculus OX=10090
Q99L45	193850000	200840000	135630000	198550000	38092	Eukaryotic translation initiation factor 2 subunit 2 OS=Mus musculus
P19001	94326000	105350000	15572000	63778000	44542	Keratin type I cytoskeletal 19 OS=Mus musculus OX=10090
P14873	55578000	62701000	25005000	50962000	270252	Microtubule-associated protein 1B OS=Mus musculus OX=10090
Q99MR8	94837000	92437000	51752000	108020000	79344	Methylcrotonoyl-CoA carboxylase subunit alpha mitochondrial OS=Mus musculus
Q8BMK4	95453000	107520000	58585000	78357000	63692	Cytoskeleton-associated protein 4 OS=Mus musculus OX=10090
P20152	95823000	99243000	22839000	75578000	53688	Vimentin OS=Mus musculus OX=10090

Q62167	114880000	164730000	82547000	150940000	73102	ATP-dependent RNA helicase DDX3X OS=Mus musculus
Q922F4	8343900	8186900	16588000	12732000	50090	Tubulin beta-6 chain OS=Mus musculus OX=10090 GN=Tubb6
Q6P542	60738000	59007000	38525000	70707000	94945	ATP-binding cassette sub-family F member 1 OS=Mus musculus
Q05920	55163000	74544000	56227000	67426000	129684	Pyruvate carboxylase mitochondrial OS=Mus musculus OX=10090
P02535	3453500	3150600	49162	1521500	57770	Keratin type I cytoskeletal 10 OS=Mus musculus OX=10090
P43274	863010000	584960000	442750000	795890000	21977	Histone H1.4 OS=Mus musculus OX=10090
Q9ERD7		20243000	0	21219000	50419	Tubulin beta-3 chain OS=Mus musculus OX=10090 GN=Tubb3
P15864	167670000	119110000	111870000	149190000	21267	Histone H1.2 OS=Mus musculus OX=10090
Q60875	48622000	39058000	23349000	41859000	111974	Rho guanine nucleotide exchange factor 2 OS=Mus musculus
P43277	145570000	106940000	98272000	132300000	22100	Histone H1.3 OS=Mus musculus OX=10090
Q61595	44249000	52203000	17032000	34875000	152592	Kinectin OS=Mus musculus OX=10090
Q3ULD5	58437000	51790000	31524000	68588000	61379	Methylcrotonoyl-CoA carboxylase beta chain mitochondrial OS=Mus musculus OX=10090
P27659	104320000	133390000	89889000	112780000	46110	60S ribosomal protein L3 OS=Mus musculus OX=10090 GN=Rpl3
P63017	41868000	34731000	24255000	24970000	70871	Heat shock cognate 71 kDa protein OS=Mus musculus OX=10090
Q8BMS1	17669000	21346000	34578000	57965000	82670	Trifunctional enzyme subunit alpha mitochondrial OS=Mus
Q7TPR4	15120000	33879000	1425200	8609300	103068	Alpha-actinin-1 OS=Mus musculus OX=10090
Q8CIE6	37819000	41685000	17589000	35891000	138432	Coatomer subunit alpha OS=Mus musculus OX=10090 GN=Copa
Q9DCV7	36698000	45947000	4010100	28997000	50709	Keratin type II cytoskeletal 7 OS=Mus musculus OX=10090
Q501J6	25935000	23979000	17688000	20574000	72400	Probable ATP-dependent RNA helicase DDX17 OS=Mus musculus
P46735	28615000	32741000	5241700	48536000	128564	Unconventional myosin-Ib OS=Mus musculus OX=10090 GN=Myo1b
Q04750	59595000	59093000	42248000	74980000	90876	DNA topoisomerase 1 OS=Mus musculus OX=10090 GN=Top1
O54774	26845000	25701000	15536000	28847000	135081	AP-3 complex subunit delta-1 OS=Mus musculus OX=10090
Q9D6Z1	202840000	177330000	134900000	180210000	64464	Nucleolar protein 56 OS=Mus musculus OX=10090 GN=Nop56

P51410	189930000	211200000	126720000	234280000	21881	60S ribosomal protein L9 OS=Mus musculus OX=10090 GN=Rpl9
Q922Q8	399840000	365670000	233200000	584080000	34877	Leucine-rich repeat-containing protein 59 OS=Mus musculus
Q03265	53169000	59770000	36247000	44192000	59753	ATP synthase subunit alpha mitochondrial OS=Mus musculus
Q5SWU9	23164000	24703000	17644000	29511000	265254	Acetyl-CoA carboxylase 1 OS=Mus musculus OX=10090 GN=Acaca
Q80UM7	65056000	61065000	41977000	61461000	91831	Mannosyl-oligosaccharide glucosidase OS=Mus
P20029	81765000	51859000	51529000	39218000	72422	Endoplasmic reticulum chaperone BiP OS=Mus musculus OX=10090
P63276	230430000	219040000	151040000	260140000	15524	40S ribosomal protein S17 OS=Mus musculus OX=10090 GN=Rps17
P39447	30467000	36360000	22353000	39970000	194740	Tight junction protein ZO-1 OS=Mus musculus OX=10090 GN=Tjp1
P16858	91130000	111710000	46016000	76714000	35810	Glyceraldehyde-3-phosphate dehydrogenase OS=Mus musculus
Q6DFW4	37536000	42189000	24841000	42656000	60343	Nucleolar protein 58 OS=Mus musculus OX=10090 GN=Nop58
Q9D8E6	71237000	92426000	56171000	76701000	47154	60S ribosomal protein L4 OS=Mus musculus OX=10090 GN=Rpl4
Q8BVY0	59727000	58657000	35033000	76607000	50421	Ribosomal L1 domain-containing protein 1 OS=Mus musculus
Q3UQ28	28227000	21536000	6849000	26460000	165102	Peroxidasin homolog OS=Mus musculus OX=10090 GN=Pxdn
V9GWY0	1489700			1133400	29939	40S ribosomal protein S4 OS=Mus musculus OX=10090
Q8K224	12691000	18354000	10873000	17499000	115418	RNA cytidine acetyltransferase OS=Mus musculus
Q8VEK3	83956000	112250000	61126000	103280000	87918	Heterogeneous nuclear ribonucleoprotein U OS=Mus musculus OX=10090 GN=Hnrnpu
Q78PY7	31124000	32655000	17859000	29668000	102088	Staphylococcal nuclease domain-containing protein 1 OS=Mus
Q3THE2	131350000	154010000	27906000	175410000	19779	Myosin regulatory light chain 12B OS=Mus musculus OX=10090
P10126	112970000	150800000	57580000	95239000	50114	Elongation factor 1-alpha 1 OS=Mus musculus OX=10090 GN=Eef1a1
P53395	52856000	58782000	33109000	19825000	53247	Lipoamide acyltransferase component of branched-chain alpha-keto acid dehydrogenase complex
F8VQC1	28506000	204960000	36980000	28066000	74657	Signal recognition particle subunit SRP72 OS=Mus musculus

Q9WTI7	21001000	21496000	5929300	26329000	121944	Unconventional myosin-Ic OS=Mus musculus OX=10090 GN=Myo1c
P26039	12648000	15449000	2037300	7424500	269819	Talin-1 OS=Mus musculus OX=10090
O54724	73541000	65068000	42419000	70836000	43954	Caveolae-associated protein 1 OS=Mus musculus OX=10090
Q99KP6	79003000	83879000	58357000	110510000	55239	Pre-mRNA-processing factor 19 OS=Mus musculus OX=10090
P43276	260510000	179610000	131460000	233430000	22576	Histone H1.5 OS=Mus musculus OX=10090
Q99KQ4	12580000	2773400	30583000	5328900	55447	Nicotinamide phosphoribosyltransferase OS=Mus musculus OX=10090 GN=Nampt
P51881	28408000	44850000	17971000	38026000	32931	ADP/ATP translocase 2 OS=Mus musculus OX=10090 GN=Slc25a5
P47911	57864000	71728000	40930000	62496000	33510	60S ribosomal protein L6 OS=Mus musculus OX=10090 GN=Rpl6
P25444	75008000	100900000	59112000	75730000	31231	40S ribosomal protein S2 OS=Mus musculus OX=10090 GN=Rps2
Q9Z0U1	25821000	28123000	17016000	28446000	131280	Tight junction protein ZO-2 OS=Mus musculus OX=10090 GN=Tjp2
P12970	69058000	79159000	50501000	70505000	29977	60S ribosomal protein L7a OS=Mus musculus OX=10090 GN=Rpl7a
P17182	27338000	59493000	21690000	22179000	47141	Alpha-enolase OS=Mus musculus OX=10090
Q99PL5	49764000	48453000	31089000	32832000	172878	Ribosome-binding protein 1 OS=Mus musculus OX=10090
P10922	360470000	282640000	264140000	386370000	20861	Histone H1.0 OS=Mus musculus OX=10090
P62301	246080000	334960000	218820000	309600000	17222	40S ribosomal protein S13 OS=Mus musculus OX=10090 GN=Rps13
Q61937	91534000	116360000	67186000	135980000	32560	Nucleophosmin OS=Mus musculus OX=10090
Q6PDG5	10117000	10272000	5918600	8871200	132604	SWI/SNF complex subunit SMARCC2 OS=Mus musculus
E9Q557	13025000	6208500	7561800	12098000	332913	Desmoplakin OS=Mus musculus OX=10090
P61255	227410000	287280000	196840000	250380000	17258	60S ribosomal protein L26 OS=Mus musculus OX=10090 GN=Rpl26
Q3TTY5	1721600	5087700		3039000	70923	Keratin type II cytoskeletal 2 epidermal OS=Mus musculus
P38647	19984000	23588000	11866000	17939000	73461	Stress-70 protein mitochondrial OS=Mus musculus OX=10090
Q3TEA8	54363000	44283000	36460000	60117000	60867	Heterochromatin protein 1-binding protein 3 OS=Mus musculus
Q80WJ7	48862000	37593000	23825000	44707000	63846	Protein LYRIC OS=Mus musculus OX=10090
P56480	21454000	41255000	15071000	24003000	56301	ATP synthase subunit beta mitochondrial OS=Mus musculus

Q9DB20	76782000	77188000	26117000	78719000	23364	ATP synthase subunit O mitochondrial OS=Mus musculus OX=10090
Q8K363	17408000	25065000	13150000	27031000	74181	ATP-dependent RNA helicase DDX18 OS=Mus musculus
P62908	112650000	130920000	67360000	112020000	26674	40S ribosomal protein S3 OS=Mus musculus OX=10090 GN=Rps3
Q5SYD0	12104000	15567000	447100	25164000	116081	Unconventional myosin-Id OS=Mus musculus OX=10090 GN=Myo1d
P62751	284580000	264620000	181660000	341270000	17695	60S ribosomal protein L23a OS=Mus musculus OX=10090 GN=Rpl23a
Q3TKT4	19471000	22057000	11002000	22049000	181426	Transcription activator BRG1 OS=Mus musculus OX=10090
Q06890	23734000	44751000	15540000	31034000	51656	Clusterin OS=Mus musculus OX=10090
Q9WVE8	23414000	20974000	29749000	42125000	55833	Protein kinase C and casein kinase substrate in neurons protein 2 OS=Mus musculus
Q9D0E1	19108000	35357000	20781000	31407000	77649	Heterogeneous nuclear ribonucleoprotein M OS=Mus musculus OX=10090 GN=Hnrnpm
Q91YQ5	9664300	17897000	6989600	11936000	68528	Dolichyl-diphosphooligosaccharide--protein glycosyltransferase
P35550	39628000	45545000	21579000	33560000	34307	rRNA 2'-O-methyltransferase fibrillarin OS=Mus
Q6A068	21062000	23337000	16185000	30955000	92190	Cell division cycle 5-like protein OS=Mus musculus OX=10090
Q9JIK5	14244000	24413000	17676000	18098000	93551	Nucleolar RNA helicase 2 OS=Mus musculus OX=10090 GN=Ddx21
F8VPU2	25837000	22053000	12774000	27446000	118875	FERM ARHGEF and pleckstrin domain-containing protein 1 OS=Mus musculus
Q9EQK5	6395000	16162000	4335700	8476400	95924	Major vault protein OS=Mus musculus
P48962	8951200	10867000	5317800	10229000	32904	ADP/ATP translocase 1 OS=Mus musculus OX=10090 GN=Slc25a4
Q5SWD9	20814000	24966000	16167000	28432000	92105	Pre-rRNA-processing protein TSR1 homolog OS=Mus musculus
P14869	25128000	44436000	16516000	36751000	34216	60S acidic ribosomal protein P0 OS=Mus musculus OX=10090
P32233	34615000	44505000	23085000	32305000	40512	Developmentally-regulated GTP-binding protein 1 OS=Mus
Q8BG05	18298000	19605000	16018000	30603000	39652	Heterogeneous nuclear ribonucleoprotein A3 OS=Mus musculus OX=10090 GN=Hnrnpa3
B2RXM2	18298000	19605000	16018000	30603000	36610	EG627828 protein OS=Mus musculus

F6YVP7	231150000	271620000	170840000	270040000	17672	Predicted gene 10260 OS=Mus musculus OX=10090
P62270	231150000	271620000	170840000	270040000	17719	40S ribosomal protein S18 OS=Mus musculus OX=10090 GN=Rps18
A0A1Y7VKY	231150000	271620000	170840000	270040000	17749	MCG116671 OS=Mus musculus OX=10090 GN=Gm11361 PE=3
P24369	53297000	54756000	26688000	39286000	23713	Peptidyl-prolyl cis-trans isomerase B OS=Mus musculus OX=10090
P11276	15825000	14464000	15686000	13901000	272536	Fibronectin OS=Mus musculus OX=10090
P13020	5527700	6184800		5451900	85942	Gelsolin OS=Mus musculus OX=10090
Q99KK2	36773000	45420000	24829000	37109000	48058	N-acylneuraminate cytidylyltransferase OS=Mus musculus
Q6ZWN5	100410000	120670000	91281000	98733000	22591	40S ribosomal protein S9 OS=Mus musculus OX=10090 GN=Rps9
Q3TIX9	25920000	25644000	13669000	21356000	65146	U4/U6.U5 tri-snRNP-associated protein 2 OS=Mus musculus
Q9QWL7	444130	0			48162	Keratin type I cytoskeletal 17 OS=Mus musculus OX=10090
Q9CPR4	52537000	67535000	49308000	63300000	21423	60S ribosomal protein L17 OS=Mus musculus OX=10090 GN=Rpl17
P29341	520880	2579900		1658000	70671	Polyadenylate-binding protein 1 OS=Mus musculus OX=10090
P17156		1271500	0		69642	Heat shock-related 70 kDa protein 2 OS=Mus musculus OX=10090
Q9DBS9	14683000	13115000	8966300	14956000	96966	Oxysterol-binding protein-related protein 3 OS=Mus musculus
P62242	87232000	101940000	58945000	93962000	24205	40S ribosomal protein S8 OS=Mus musculus OX=10090 GN=Rps8
Q9DBG3	16328000	27766000	8407000	18699000	104583	AP-2 complex subunit beta OS=Mus musculus OX=10090 GN=Ap2b1
P58771	12536000	7883100	236400	15428000	32681	Tropomyosin alpha-1 chain OS=Mus musculus OX=10090 GN=Tpm1
P62843	124820000	138150000	70323000	180930000	17040	40S ribosomal protein S15 OS=Mus musculus OX=10090 GN=Rps15
P11103	4774400	10572000	7359500	8623200	113100	Poly [ADP-ribose] polymerase 1 OS=Mus musculus OX=10090
P63325	132150000	116870000	91349000	126250000	18916	40S ribosomal protein S10 OS=Mus musculus OX=10090 GN=Rps10
P62900	115980000	125500000	122580000	147840000	14463	60S ribosomal protein L31 OS=Mus musculus OX=10090 GN=Rpl31
P14148	51756000	69645000	48698000	55342000	31420	60S ribosomal protein L7 OS=Mus musculus OX=10090 GN=Rpl7

Q8BP47	11434000	14499000	5490700	10750000	64279	Asparagine--tRNA ligase cytoplasmic OS=Mus musculus OX=10090
P97496	10970000	13298000	6798600	11518000	122890	SWI/SNF complex subunit SMARCC1 OS=Mus musculus
P17225	21040000	22867000	10515000	18619000	56478	Polypyrimidine tract-binding protein 1 OS=Mus musculus
P04104		1479500		220150	65606	Keratin type II cytoskeletal 1 OS=Mus musculus OX=10090
P57780	590520	2216200		353410	104977	Alpha-actinin-4 OS=Mus musculus OX=10090
P58252	6286800	14635000	3727300	7355500	95314	Elongation factor 2 OS=Mus musculus
P43275	49300000	26976000	27157000	44782000	21785	Histone H1.1 OS=Mus musculus OX=10090
Q02257	15535000	7107800	9465500	10405000	81801	Junction plakoglobin OS=Mus musculus OX=10090 GN=Jup
Q9Z1Q9	14428000	13927000	10030000	10812000	140215	Valine--tRNA ligase OS=Mus musculus OX=10090 GN=Vars
P70670	41998000	43269000	20045000	38156000	220497	Nascent polypeptide-associated complex subunit alpha muscle-specific form OS=Mus
Q9DBE9	4583700	5287800	3231200	6556700	95532	pre-rRNA 2'-O-ribose RNA methyltransferase FTSJ3 OS=Mus musculus OX=10090
P17427	6424400	11260000	1714800	6827800	104016	AP-2 complex subunit alpha-2 OS=Mus musculus OX=10090
Q60997	2372300	190310	13713000	34213000	226814	Deleted in malignant brain tumors 1 protein OS=Mus musculus
Q8BGZ7	679000				59741	Keratin type II cytoskeletal 75 OS=Mus musculus OX=10090
Q64525	190670000	186600000	139870000	191240000	13920	Histone H2B type 2-B OS=Mus musculus OX=10090
Q1HFZ0	18068000	20791000	12379000	17679000	85452	tRNA (cytosine(34)-C(5))-methyltransferase OS=Mus musculus
P62264	35571000	36899000	33483000	38647000	16273	40S ribosomal protein S14 OS=Mus musculus OX=10090 GN=Rps14
Q9CQ19	1688900	1763600		1840700	19854	Myosin regulatory light polypeptide 9 OS=Mus musculus OX=10090
Q9JHJ0	24544000	20854000		37825000	39503	Tropomodulin-3 OS=Mus musculus
P28740	10956000	13649000	10082000	19596000	79756	Kinesin-like protein KIF2A OS=Mus musculus OX=10090
P17426	3845600	4998500	1585100	6969800	107664	AP-2 complex subunit alpha-1 OS=Mus musculus OX=10090
Q9QXS1	5933700	560890	900810	3294100	534193	Plectin OS=Mus musculus OX=10090
P62918	40696000	45696000	31262000	40922000	28025	60S ribosomal protein L8 OS=Mus musculus OX=10090 GN=Rpl8

P52480	2352500	24077000	5928000	4081700	57845	Pyruvate kinase PKM OS=Mus musculus OX=10090 GN=Pkm
P97315	19248000	19862000	13054000	14866000	20583	Cysteine and glycine-rich protein 1 OS=Mus musculus OX=10090
Q9CXS4	18921000	19062000	15061000	21318000	27541	Centromere protein V OS=Mus musculus OX=10090 GN=Cenpv
P62849	76522000	86497000	62491000	83898000	15423	40S ribosomal protein S24 OS=Mus musculus OX=10090 GN=Rps24
P09405	11727000	9247100	9413700	9917100	76723	Nucleolin OS=Mus musculus OX=10090
Q8VED5	0				57552	Keratin type II cytoskeletal 79 OS=Mus musculus OX=10090
Q6PHQ9	794940	1275800	815610	377850	72242	Polyadenylate-binding protein OS=Mus musculus OX=10090
P07356	12366000	17633000	7334300	5053200	38676	Annexin A2 OS=Mus musculus OX=10090
P62281	48076000	57273000	32009000	42606000	18431	40S ribosomal protein S11 OS=Mus musculus OX=10090 GN=Rps11
E9Q1Z0	551210	839140	38526	942100	58224	Keratin 90 OS=Mus musculus OX=10090
P97461	94657000	91911000	59178000	112420000	22889	40S ribosomal protein S5 OS=Mus musculus OX=10090 GN=Rps5
Q8CGF7	11273000	13924000	6342600	9963500	123788	Transcription elongation regulator 1 OS=Mus musculus OX=10090
P61979	14176000	19386000	10604000	13220000	50976	Heterogeneous nuclear ribonucleoprotein K OS=Mus musculus OX=10090 GN=Hnrnpk
D3Z2H9	24974000	18067000		29818000	28992	Tropomyosin 3 related sequence 7 OS=Mus musculus OX=10090
Q9CZM2	9261400	12812000	7222300	12084000	24146	60S ribosomal protein L15 OS=Mus musculus OX=10090 GN=Rpl15
P62827	23265000	26442000	16194000	27687000	24423	GTP-binding nuclear protein Ran OS=Mus musculus OX=10090
Q91VR5	7586200	14055000	4520200	9775600	82500	ATP-dependent RNA helicase DDX1 OS=Mus musculus OX=10090
P11499	2918100	11816000	2149500	1357400	83281	Heat shock protein HSP 90-beta OS=Mus musculus OX=10090
Q8BMA6	16072000	25594000	12034000	21727000	70574	Signal recognition particle subunit SRP68 OS=Mus musculus
Q8VHE0	21736000	17263000	12424000	15709000	87870	Translocation protein SEC63 homolog OS=Mus musculus
P61358	43684000	50316000	23546000	57503000	15798	60S ribosomal protein L27 OS=Mus musculus OX=10090 GN=Rpl27
P84099	44101000	50772000	36957000	57420000	23466	60S ribosomal protein L19 OS=Mus musculus OX=10090 GN=Rpl19

Q9JJ28	8403300	8421600	927920	10063000	144803	Protein flightless-1 homolog OS=Mus musculus OX=10090
O70551	9911300	10627000	6825600	12520000	73089	SRSF protein kinase 1 OS=Mus musculus OX=10090 GN=Srpk1
Q9CXW4	54458000	58699000	32183000	49943000	20252	60S ribosomal protein L11 OS=Mus musculus OX=10090 GN=Rpl11
P80314	2552800	4247500	1565000	2823300	57477	T-complex protein 1 subunit beta OS=Mus musculus OX=10090
Q8CGC7	2726700	4116200	2099000	4415700	170078	Bifunctional glutamate/proline--tRNA ligase OS=Mus musculus
Q6ZWV7	181690000	155480000	111840000	195160000	14553	60S ribosomal protein L35 OS=Mus musculus OX=10090 GN=Rpl35
Q3UV17	268930				62845	Keratin type II cytoskeletal 2 oral OS=Mus musculus
P35979	22705000	28981000	14053000	17372000	17805	60S ribosomal protein L12 OS=Mus musculus OX=10090 GN=Rpl12
Q64512	4221600	3707800	3129800	5635200	270332	Tyrosine-protein phosphatase non-receptor type 13 OS=Mus
P63101	176670	2152100	276760	946790	27771	14-3-3 protein zeta/delta OS=Mus musculus OX=10090 GN=Ywhaz
Q80UM3	6544800	9281200	4952000	7182900	100961	N-alpha-acetyltransferase 15 NatA auxiliary subunit OS=Mus
P62754	50290000	66497000	28840000	50358000	28681	40S ribosomal protein S6 OS=Mus musculus OX=10090 GN=Rps6
Q8BGS1	12103000	10698000	6959500	18652000	81635	Band 4.1-like protein 5 OS=Mus musculus OX=10090 GN=Epb41l5
O35737	608320	0	360680	0	49199	Heterogeneous nuclear ribonucleoprotein H OS=Mus musculus OX=10090 GN=Hnrnph1
P47963	77565000	70597000	68941000	76594000	24305	60S ribosomal protein L13 OS=Mus musculus OX=10090 GN=Rpl13
Q9CQE8	2559700	5087400	1942700	7393500	28152	RNA transcription translation and transport factor protein OS=Mus musculus OX=10090
P30999	6351300	8231100	2544500	6366000	104925	Catenin delta-1 OS=Mus musculus OX=10090
Q7TMK9	3515300	11779000	2431200	3645200	69633	Heterogeneous nuclear ribonucleoprotein Q OS=Mus musculus OX=10090 GN=Syncrip
Q64467	1991700	1672000	1235200	1942000	47657	Glyceraldehyde-3-phosphate dehydrogenase testis-specific OS=Mus musculus OX=10090
Q61749	14648000	9193600	6117300	8774700	57624	Translation initiation factor eIF-2B subunit delta OS=Mus musculus
Q9CZX8	62184000	45100000	49198000	82762000	16085	40S ribosomal protein S19 OS=Mus musculus OX=10090 GN=Rps19

P16546	2590500	2696600	1409600	1367100	284596	Spectrin alpha chain non-erythrocytic 1 OS=Mus musculus
Q8BMF4	1558500	11439000	8219100	7102700	67942	Dihydrolipoyllysine-residue acetyltransferase component of pyruvate dehydrogenase complex mitochondrial OS=Mus
Q9Z1T1	6449400	8891900	4305600	5283500	122740	AP-3 complex subunit beta-1 OS=Mus musculus OX=10090
P62911	12872000	22534000	11617000	15023000	15860	60S ribosomal protein L32 OS=Mus musculus OX=10090 GN=Rpl32
Q8CCP0	7363000	6302600	4619400	5006100	121188	Nuclear export mediator factor Nemf OS=Mus musculus OX=10090
Q6P5B0	3849500	4051000	1828800	4823300	143131	RRP12-like protein OS=Mus musculus
E9Q4Z2	430070				275748	Acetyl-CoA carboxylase 2 OS=Mus musculus OX=10090 GN=Acacb
O70310	10285000	13064000	8399700	6866000	56888	Glycylpeptide N-tetradecanoyltransferase 1 OS=Mus musculus OX=10090 GN=Nmt1
Q91VR2	9868200	13457000	6527100	12132000	32886	ATP synthase subunit gamma mitochondrial OS=Mus musculus
Q99JY0	23902000	23985000	12862000	21636000	51386	Trifunctional enzyme subunit beta mitochondrial OS=Mus
Q6NXH9				634630	58911	Keratin type II cytoskeletal 73 OS=Mus musculus OX=10090
Q8BKC5	4275000	8978400	2907500	7594900	123591	Importin-5 OS=Mus musculus OX=10090
Q3TDQ1	11826000	14864000	10392000	10671000	93246	Dolichyl-diphosphooligosaccharide--protein glycosyltransferase
P53026	17935000	27368000	10339000	17988000	24916	60S ribosomal protein L10a OS=Mus musculus OX=10090 GN=Rpl10a
Q6IFZ9	2107700	1172200		1294100	54747	Keratin type II cytoskeletal 74 OS=Mus musculus OX=10090
P62960	12197000	46227000	14153000	28770000	35730	Nuclease-sensitive element-binding protein 1 OS=Mus musculus
Q9EPU4	4651000	5846900	2822700	5633300	160818	Cleavage and polyadenylation specificity factor subunit 1 OS=Mus musculus
Q6ZWV3	6633100	12073000	3285900	8364100	24604	60S ribosomal protein L10 OS=Mus musculus OX=10090 GN=Rpl10
Q921N6	8217400	8307500	6810700	11032000	85939	Probable ATP-dependent RNA helicase DDX27 OS=Mus musculus
Q3TIV5	13588000	13042000	11574000	10148000	48327	Zinc finger CCCH domain-containing protein 15 OS=Mus
Q64511	1013900	5507000	2965400	6738200	181908	DNA topoisomerase 2-beta OS=Mus musculus OX=10090 GN=Top2b

Q9Z2X1	7934200	10335000	6862300	8763200	45730	Heterogeneous nuclear ribonucleoprotein F OS=Mus musculus
Q922V4	8316100	8035000	5120700	9895100	56938	Pleiotropic regulator 1 OS=Mus musculus OX=10090 GN=Plrg1
P22777	6104300	8614000	6566000	11982000	45170	Plasminogen activator inhibitor 1 OS=Mus musculus OX=10090
Q60605	9156600	10370000	2540900	18321000	16930	Myosin light polypeptide 6 OS=Mus musculus OX=10090 GN=Myl6
P47962	11178000	20122000	13572000	11749000	34401	60S ribosomal protein L5 OS=Mus musculus OX=10090 GN=Rpl5
Q03350	331960	0	137240	0	129882	Thrombospondin-2 OS=Mus musculus
Q8BP67	56474000	58289000	37160000	51040000	17779	60S ribosomal protein L24 OS=Mus musculus OX=10090 GN=Rpl24
P62717	3545600	16748000	2548900	11358000	20732	60S ribosomal protein L18a OS=Mus musculus OX=10090 GN=Rpl18a
O70318	3734200	6346200	3453300	14235000	109940	Band 4.1-like protein 2 OS=Mus musculus OX=10090 GN=Epb41l2
Q6PGC1	1688000	2759500	1155400	2498000	153975	ATP-dependent RNA helicase DHX29 OS=Mus musculus
P62852	65597000	71951000	51277000	71479000	13742	40S ribosomal protein S25 OS=Mus musculus OX=10090 GN=Rps25
P84091	7769500	11294000	13670000	9727100	49655	AP-2 complex subunit mu OS=Mus musculus OX=10090 GN=Ap2m1
O70503	2247100	7013700	0	2479200	34742	Very-long-chain 3-oxoacyl-CoA reductase OS=Mus musculus OX=10090
P57776	4868600	16314000	5219800	10101000	31293	Elongation factor 1-delta OS=Mus musculus OX=10090 GN=Eef1d
Q9CR57	34740000	41925000	20544000	27626000	23564	60S ribosomal protein L14 OS=Mus musculus OX=10090 GN=Rpl14
Q9Z315	2668400	3475300	1449600	4786700	90885	U4/U6.U5 tri-snRNP-associated protein 1 OS=Mus musculus
P62141	2760900	3313100	1342000	2389000	37187	Serine/threonine-protein phosphatase PP1-beta catalytic subunit OS=Mus musculus
P14131	15520000	17129000	13389000	20535000	16445	40S ribosomal protein S16 OS=Mus musculus OX=10090 GN=Rps16
P35980	40603000	55366000	36687000	24406000	21645	60S ribosomal protein L18 OS=Mus musculus OX=10090 GN=Rpl18
Q61103	7936200	6564400	4406300	6146900	44230	Zinc finger protein ubi-d4 OS=Mus musculus OX=10090 GN=Dpf2
O09167	21955000	25139000	10772000	20671000	18562	60S ribosomal protein L21 OS=Mus musculus OX=10090 GN=Rpl21

P19253	27486000	38742000	23363000	21077000	23464	60S ribosomal protein L13a OS=Mus musculus OX=10090 GN=Rpl13a
S4R1R7	0				21992	MCG50795 OS=Mus musculus OX=10090
Q9D824	3212600	2119800	2591900	4601400	64959	Pre-mRNA 3'-end-processing factor FIP1 OS=Mus musculus
Q99020	9420900	11197000	7111200	1738200	30831	Heterogeneous nuclear ribonucleoprotein A/B OS=Mus musculus OX=10090 GN=Hnrnpab
O35286	5943700	10791000	1454600	8665400	91007	Pre-mRNA-splicing factor ATP-dependent RNA helicase DHX15 OS=Mus musculus
P09103	1789500	5381500	1325000	2175400	57059	Protein disulfide-isomerase OS=Mus musculus OX=10090
P29788	840320			613710	54849	Vitronectin OS=Mus musculus OX=10090
P18760	14002000	21616000	8413500	13570000	18560	Cofilin-1 OS=Mus musculus OX=10090
O54941	9680600	6734600	5125300	8359300	46638	SWI/SNF-related matrix-associated actin-dependent regulator of chromatin subfamily E member 1 OS=Mus
V9GXQ2		6111000			17596	Predicted gene 17087 OS=Mus musculus OX=10090
A2AT37	2779400	3031300	1292700	3205400	147552	UPF2 regulator of nonsense transcripts homolog (Yeast)
Q9R112	5595900	13197000	4504300	9383900	50282	Sulfide:quinone oxidoreductase mitochondrial OS=Mus
Q99LF4	5397900	9344800	3144600	8589900	55249	tRNA-splicing ligase RtcB homolog OS=Mus musculus OX=10090
Q9R1R2	2130500	3118600	117610	3005900	80775	Tripartite motif-containing protein 3 OS=Mus musculus
Q8BFR5	3819100	28215000	23050000	32406000	49508	Elongation factor Tu mitochondrial OS=Mus musculus OX=10090
P21981	8962200	11159000	1886300	9022800	77061	Protein-glutamine gamma-glutamyltransferase 2 OS=Mus musculus
P07724	1830300	172340	130410	2167800	68693	Serum albumin OS=Mus musculus OX=10090
Q6IRU2	7670400	6026600		11524000	28468	Tropomyosin alpha-4 chain OS=Mus musculus OX=10090 GN=Tpm4
O70133	2612800	4210500	2081600	4462500	149474	ATP-dependent RNA helicase A OS=Mus musculus OX=10090
Q8VH51	7110600	3027900	7536000	4557800	59407	RNA-binding protein 39 OS=Mus musculus OX=10090 GN=Rbm39
Q8BKX1	7004200	7045600	5034600	6688400	59237	Brain-specific angiogenesis inhibitor 1-associated protein 2

P07901	694480	2577200	412000	520900	84788	Heat shock protein HSP 90-alpha OS=Mus musculus OX=10090
P58774	302350			695960	32837	Tropomyosin beta chain OS=Mus musculus OX=10090 GN=Tpm2
Q99JR8	3233100	3662100	2129100	3452100	59085	SWI/SNF-related matrix-associated actin-dependent regulator of chromatin subfamily D member 2 OS=Mus
Q61696				86253	70079	Heat shock 70 kDa protein 1A OS=Mus musculus OX=10090
P17879				86253	70176	Heat shock 70 kDa protein 1B OS=Mus musculus OX=10090
E9Q0F0			158460		112265	Keratin 78 OS=Mus musculus OX=10090
P60766	7639800	12262000	5697900	14290000	21259	Cell division control protein 42 homolog OS=Mus musculus
Q61768	1626200	4232700	1427600	929610	109551	Kinesin-1 heavy chain OS=Mus musculus OX=10090 GN=Kif5b
Q8VHX6	13929000	118530		180590	291117	Filamin-C OS=Mus musculus OX=10090
Q9Z1R9	658820000	210880000	417310000	381710000	26135	MCG124046 OS=Mus musculus OX=10090
Q9Z2D6	4455700	3957900	1786800	3979600	52308	Methyl-CpG-binding protein 2 OS=Mus musculus OX=10090
Q99NH0	1249500	1236800	668870	1594600	274211	Ankyrin repeat domain-containing protein 17 OS=Mus musculus
D3YTY6		638270		0	17224	Predicted gene 6096 OS=Mus musculus OX=10090 GN=Gm6096
P27661	41320000	36944000	33539000	38675000	15143	Histone H2AX OS=Mus musculus OX=10090
P62983	78684000	64280000	51205000	75335000	17951	Ubiquitin-40S ribosomal protein S27a OS=Mus musculus OX=10090
P0CG49	78684000	64280000	51205000	75335000	34369	Polyubiquitin-B OS=Mus musculus OX=10090
P0CG50	78684000	64280000	51205000	75335000	82550	Polyubiquitin-C OS=Mus musculus OX=10090
A0A0A6YW6	78684000	64280000	51205000	75335000	8728	MCG23377 isoform CRA_b OS=Mus musculus OX=10090
P62984	78684000	64280000	51205000	75335000	14728	Ubiquitin-60S ribosomal protein L40 OS=Mus musculus OX=10090
P07744	0	296190	94252		56283	Keratin type II cytoskeletal 4 OS=Mus musculus OX=10090
Q792Z1	109390000	115610000	173600000	179060000	26221	MCG140784 OS=Mus musculus OX=10090
Q8VCR2	6594100	8476100	2847400	7619900	33458	17-beta-hydroxysteroid dehydrogenase 13 OS=Mus musculus
Q00PI9	2939100	7359800	5360300	7060800	84940	Heterogeneous nuclear ribonucleoprotein U-like protein 2 OS=Mus musculus OX=10090

P70333		201220			49280	Heterogeneous nuclear ribonucleoprotein H2 OS=Mus musculus OX=10090 GN=Hnrnph2
P62137				656610	37540	Serine/threonine-protein phosphatase PP1-alpha catalytic subunit OS=Mus musculus
Q99LG0	4715400	2393500	1767900	5754900	93434	Ubiquitin carboxyl-terminal hydrolase 16 OS=Mus musculus
Q68FD5	219860	4841100	323990	593880	191555	Clathrin heavy chain 1 OS=Mus musculus OX=10090 GN=Cltc
Q9Z2H5	1680300	2079100	1706200	3843700	98315	Band 4.1-like protein 1 OS=Mus musculus OX=10090 GN=Epb41l1
Q922K7	5565100	5070300	3066700	3992100	86752	Probable 28S rRNA (cytosine-C(5))-methyltransferase
P11983	3799600	7503000	1581900	4546900	60449	T-complex protein 1 subunit alpha OS=Mus musculus OX=10090
P19157	7039900	15599000	2581900	959740	23609	Glutathione S-transferase P 1 OS=Mus musculus OX=10090 GN=Gstp1
Q9DB77	1171700	2128900	556150	1772300	48235	Cytochrome b-c1 complex subunit 2 mitochondrial OS=Mus
Q9QZQ8	3153800	3951600	849900	3886400	39735	Core histone macro-H2A.1 OS=Mus musculus OX=10090
Q9WV92	2176000	4364000	1305900	6960600	103338	Band 4.1-like protein 3 OS=Mus musculus OX=10090 GN=Epb41l3
P14115	42683000	51377000	29361000	43911000	16605	60S ribosomal protein L27a OS=Mus musculus OX=10090 GN=Rpl27a
Q9D0I9	2497200	6137700	1695600	2280800	75674	Arginine--tRNA ligase cytoplasmic OS=Mus musculus OX=10090
P62259	222390	1144900	83351	911850	29174	14-3-3 protein epsilon OS=Mus musculus OX=10090 GN=Ywhae
P21107	2220400	2285300		2351700	32994	Tropomyosin alpha-3 chain OS=Mus musculus OX=10090 GN=Tpm3
Q8R0X7	2913500	5798600	1462700	5835500	63677	Sphingosine-1-phosphate lyase 1 OS=Mus musculus OX=10090
P57784	8365500	7030100	3232900	5130100	28357	U2 small nuclear ribonucleoprotein A' OS=Mus musculus
Q3V132	66684				35258	ADP/ATP translocase 4 OS=Mus musculus OX=10090
Q6P9Q4	1289400	1049900	330790	3802900	129600	FH1/FH2 domain-containing protein 1 OS=Mus musculus
Q9D4E6	0		146090		71001	Polyadenylate-binding protein OS=Mus musculus OX=10090
Q9D287	1878500	4069300	3547300	6702200	26131	Pre-mRNA-splicing factor SPF27 OS=Mus musculus OX=10090

Q8BK63	4067100	3164300	2702900	3205900	38915	Casein kinase I isoform alpha OS=Mus musculus OX=10090 GN=Csnk1a1
P35700	18218000	17573000	8010200	15273000	22176	Peroxiredoxin-1 OS=Mus musculus
Q9D8N0	7480000	10994000	3020200	7298900	50061	Elongation factor 1-gamma OS=Mus musculus OX=10090
P62806	7016700	9335300	5466700	9394000	11367	Histone H4 OS=Mus musculus OX=10090
P62267	8209100	14350000	8757800	12586000	15808	40S ribosomal protein S23 OS=Mus musculus OX=10090 GN=Rps23
E9Q634	2952000	3509400	1522200	3681400	126818	Unconventional myosin-Ie OS=Mus musculus OX=10090 GN=Myo1e
P60843	2939400	7399000	1593100	2943700	46154	Eukaryotic initiation factor 4A-I OS=Mus musculus OX=10090
Q80X41	5165500	3605100	989360	2529900	49741	Serine/threonine-protein kinase VRK1 OS=Mus musculus OX=10090
P14206	5149200	4196400	2274200	3982200	32838	40S ribosomal protein SA OS=Mus musculus OX=10090 GN=Rpsa
Q810D6	1681600	1942000	605060	3568200	49224	Glutamate-rich WD repeat-containing protein 1 OS=Mus musculus
Q02248	488890	1181700	376170	683690	85471	Catenin beta-1 OS=Mus musculus OX=10090
P39876	8726500	9211300	4544500	9018700	24182	Metalloproteinase inhibitor 3 OS=Mus musculus OX=10090
Q922P9	3371600	2383300	1944400	4165000	59716	Putative oxidoreductase GLYR1 OS=Mus musculus OX=10090
Q6IFZ8	8803000	7607500		5084200	60228	MCG1050941 OS=Mus musculus OX=10090
Q922B2	12803000	10498000	2972400	1641100	57147	Aspartate--tRNA ligase cytoplasmic OS=Mus musculus OX=10090
Q6IME9	0			1251200	56750	Keratin type II cytoskeletal 72 OS=Mus musculus OX=10090
Q6NSQ7	2474000	2078200	781740	2411900	54023	Protein LTV1 homolog OS=Mus musculus OX=10090 GN=Ltv1
Q3U0S6	2936900	3847900	1261900	2000900	103558	Ras-interacting protein 1 OS=Mus musculus OX=10090 GN=Rasip1
Q6P4T2	747820	746000	188990	1242700	244544	U5 small nuclear ribonucleoprotein 200 kDa helicase OS=Mus
Q9CQY5	5479900	5238300	2383300	4772300	37970	Magnesium transporter protein 1 OS=Mus musculus OX=10090
Q61033	5023200	5511700	3510400	4835500	75168	Lamina-associated polypeptide 2 isoforms alpha/zeta OS=Mus
D3Z6P0	4261500	6633400	2377800	3377700	58317	Protein disulfide-isomerase A2 OS=Mus musculus OX=10090
Q8CCF0	2637500	4217600	332800	1298300	55430	U4/U6 small nuclear ribonucleoprotein Prp31 OS=Mus musculus

Q2KN98		486270		4830500	124488	Cytospin-A OS=Mus musculus OX=10090
Q9R0P5	3353600	5794100	918810	4755400	18522	Destrin OS=Mus musculus OX=10090
Q9EQ61	2168200	1128900	1560700	2910400	67796	Pescadillo homolog OS=Mus musculus
Q3TJD7	3101700	5920500	1567100	3149300	50119	PDZ and LIM domain protein 7 OS=Mus musculus OX=10090
Q9CR62	780740	2421900	476910	1957000	34155	Mitochondrial 2-oxoglutarate/malate carrier protein OS=Mus
Q9JI13	3279600	3781200	1605700	6464400	53399	Something about silencing protein 10 OS=Mus musculus
Q64152	5526700	5820000	2338600	5197400	22031	Transcription factor BTF3 OS=Mus musculus OX=10090 GN=Btf3
Q80UU2	2132600	2210200	1586600	2113600	31129	Ribonuclease P protein subunit p38 OS=Mus musculus OX=10090
Q9Z204	1826900	4833800	1282600	2160400	34385	Heterogeneous nuclear ribonucleoproteins C1/C2 OS=Mus musculus OX=10090
Q9DAW6	1458700	1243500	1312700	1997400	58370	U4/U6 small nuclear ribonucleoprotein Prp4 OS=Mus musculus
Q9JMH9	401720	1395600		1546900	232753	Unconventional myosin-XVIIIa OS=Mus musculus OX=10090
Q9D8X2	12161000	7965100	11899000	8748000	25346	Coiled-coil domain-containing protein 124 OS=Mus musculus
Q5F2E8	5577600	5708600	4614500	5841700	116050	Serine/threonine-protein kinase TAO1 OS=Mus musculus OX=10090
Q9QYJ0	3299900	5477200	2172700	4786300	45746	DnaJ homolog subfamily A member 2 OS=Mus musculus OX=10090
P41105	9621300	8841100	8015300	12567000	15733	60S ribosomal protein L28 OS=Mus musculus OX=10090 GN=Rpl28
Q921I2	2844500	2236000	2556800	2534700	64861	Kelch domain-containing protein 4 OS=Mus musculus OX=10090
Q8VHM5	1735100	2339700	1309100	1565200	70888	Heterogeneous nuclear ribonucleoprotein R OS=Mus musculus
P32067	2141600	2900300	1125100	2388500	47756	Lupus La protein homolog OS=Mus musculus OX=10090
P08249	14681000	14379000	11528000	15725000	35611	Malate dehydrogenase mitochondrial OS=Mus musculus OX=10090
Q8BP48	4058100	4162700	3379000	4364900	43221	Methionine aminopeptidase 1 OS=Mus musculus
E9QA15	7452000	5198100	1636100	3760800	89274	Caldesmon 1 OS=Mus musculus OX=10090
E9PUR0		2386400			269675	Ankyrin repeat and KH domain-containing 1 OS=Mus musculus
Q99PV0	121370	480720	0	741580	273615	Pre-mRNA-processing-splicing factor 8 OS=Mus musculus OX=10090

B2RY56	986710	815570	245330	806090	99552	RNA-binding protein 25 OS=Mus musculus OX=10090 GN=Rbm25
Q6PDQ2	3609000	3208600	1566400	1817300	217749	Chromodomain-helicase-DNA-binding protein 4 OS=Mus musculus
P47757	3583300	5060700		4558500	31345	F-actin-capping protein subunit beta OS=Mus musculus OX=10090
Q99JF8	5529200	3066800	4332100	4680100	59697	PC4 and SFRS1-interacting protein OS=Mus musculus
Q9CX86	406670	1009600	861600	707960	30530	Heterogeneous nuclear ribonucleoprotein A0 OS=Mus musculus OX=10090 GN=Hnrnpa0
Q99M73		300910			64983	Keratin type II cuticular Hb4 OS=Mus musculus OX=10090 GN=Krt84
Q921M3	2682700	3830200	2351000	2674200	135550	Splicing factor 3B subunit 3 OS=Mus musculus OX=10090
Q922H2	2101700	1747200	1053400	1944300	47923	[Pyruvate dehydrogenase (acetyl-transferring)] kinase isozyme 3 mitochondrial OS=Mus
P28352	5184100	2939800	2356400	3932600	35490	DNA-(apurinic or apyrimidinic site) lyase OS=Mus musculus
Q3U9G9	1210000	1622800	867630	2357900	71440	Delta(14)-sterol reductase OS=Mus musculus OX=10090
J3QQ02			500630		16571	Predicted gene 2056 OS=Mus musculus OX=10090 GN=Gm2056
Q9D1M7	13175000	15278000	5259700	12699000	22137	Peptidyl-prolyl cis-trans isomerase FKBP11 OS=Mus musculus
P47754	3799600	5086300		5757300	32967	F-actin-capping protein subunit alpha-2 OS=Mus musculus OX=10090
P01027	259470000	238640000	156490000	268920000	186483	Complement C3 OS=Mus musculus
P42669	2724500	2462200	3896500	3177100	34884	Transcriptional activator protein Pur-alpha OS=Mus musculus
P84228	49755000	49282000	29232000	36190000	15388	Histone H3.2 OS=Mus musculus OX=10090 GN=Hist2h3c2 PE=1
P68433	49755000	49282000	29232000	36190000	15404	Histone H3.1 OS=Mus musculus OX=10090
P47915	9138800	10975000	8804300	7313000	17587	60S ribosomal protein L29 OS=Mus musculus OX=10090 GN=Rpl29
Q3ULL6	998380	1445200	526730	1210100	57066	UPF3 regulator of nonsense transcripts homolog B (yeast)
Q9R0G8	3365600	3520900	1558200	3289300	163647	Nik-related protein kinase OS=Mus musculus OX=10090
P61982		131400	259980		28303	14-3-3 protein gamma OS=Mus musculus OX=10090 GN=Ywhag
P68254		112090		530120	27778	14-3-3 protein theta OS=Mus musculus

Q921F2	1401700	1792900	89347	889510	44548	TAR DNA-binding protein 43 OS=Mus musculus OX=10090
O70311	483030	810780	494160	462210	60484	Glycylpeptide N-tetradecanoyltransferase 2 OS=Mus musculus OX=10090 GN=Nmt2
P47738	1847700	4584500		1009100	56538	Aldehyde dehydrogenase mitochondrial OS=Mus musculus OX=10090
Q3URQ0	2306600	1791700	1121100	2745800	105209	Testis-expressed protein 10 OS=Mus musculus OX=10090 GN=Tex10
Q5U3K5	13447000	1124700	7082500	1928000	79831	Rab-like protein 6 OS=Mus musculus
P68040	4513300	4833600	2504400	3691800	35077	Receptor of activated protein C kinase 1 OS=Mus musculus
P21271	419820	1761300		2911900	210569	Unconventional myosin-Vb OS=Mus musculus OX=10090 GN=Myo5b
Q9Z1M8	2991400	3490300	1541400	2190400	65616	Protein Red OS=Mus musculus OX=10090
P62821	1066400	1736300	648460	1379200	22678	Ras-related protein Rab-1A OS=Mus musculus OX=10090 GN=Rab1A
P19324	0	1467800			46534	Serpin H1 OS=Mus musculus OX=10090
P68510		749550			28212	14-3-3 protein eta OS=Mus musculus
O08784	4859000	2395900	2496000	3682000	135001	Treacle protein OS=Mus musculus OX=10090
Q9DBR7	2526400	2211800	1461000	3058100	114996	Protein phosphatase 1 regulatory subunit 12A OS=Mus musculus
Q9Z2N8	5458200	443010	4893900	6249200	47448	Actin-like protein 6A OS=Mus musculus OX=10090 GN=Actl6a
Q9ESX5	1324400	3296100	1543700	2415500	57402	H/ACA ribonucleoprotein complex subunit DKC1
Q3UPH7	789780	457240	160560	621460	165132	Rho guanine nucleotide exchange factor 40 OS=Mus musculus
O08663	1001400	409120			52922	Methionine aminopeptidase 2 OS=Mus musculus
Q01320	825070				172789	DNA topoisomerase 2-alpha OS=Mus musculus OX=10090 GN=Top2a
Q61087	1158100	4504700	1692500	1947300	128900	Laminin subunit beta-3 OS=Mus musculus OX=10090 GN=Lamb3
Q62261	118290	706520	0	0	274221	Spectrin beta chain non-erythrocytic 1 OS=Mus musculus OX=10090
Q8C0C7	1795100	2101300	1245500	2185200	57599	Phenylalanine--tRNA ligase alpha subunit OS=Mus musculus
P62855	4763600	2336700	4717300	5233900	13015	40S ribosomal protein S26 OS=Mus musculus OX=10090 GN=Rps26
Q8VEM8	3576300	5460000	2640800	5048800	39632	Phosphate carrier protein mitochondrial OS=Mus musculus OX=10090

Q9CPN9			809420		26422	RIKEN cDNA 2210010C04 gene OS=Mus musculus
O08582	771020	1450800		428780	72301	GTP-binding protein 1 OS=Mus musculus OX=10090 GN=Gtpbp1
Q8VHZ7	1312500	2086200	1716300	1790300	33656	U3 small nucleolar ribonucleoprotein protein IMP4 OS=Mus musculus
Q925I1	673640	997200	801980	445680	66742	ATPase family AAA domain-containing protein 3 OS=Mus
Q9DCR2	4133600	3591300	2253500	2119600	21732	AP-3 complex subunit sigma-1 OS=Mus musculus OX=10090
Q8CI11	1244200	2593600	900650	495380	60787	Guanine nucleotide-binding protein-like 3 OS=Mus musculus
Q9WVA3	1902300	1639400	718700	1218200	36955	Mitotic checkpoint protein BUB3 OS=Mus musculus OX=10090
P06151		3545300			36499	L-lactate dehydrogenase A chain OS=Mus musculus OX=10090
B2RY53			0		21973	EG620155 protein OS=Mus musculus
P97434	427010	1050900		1561200	116408	Myosin phosphatase Rho-interacting protein OS=Mus musculus
P48193	318160	203700	1038800	3928200	95911	Protein 4.1 OS=Mus musculus OX=10090
Q9CZN7	3614000	4319700	476160	1106800	55759	Serine hydroxymethyltransferase mitochondrial OS=Mus
Q8VIJ6	294570	1669700		363970	75442	Splicing factor proline- and glutamine-rich OS=Mus musculus
P11214		2177800	1794300	2339600	63097	Tissue-type plasminogen activator OS=Mus musculus OX=10090
Q8R0W0	2350700	815250		2435200	724747	Epiplakin OS=Mus musculus OX=10090
P47753	4618300	4513300		4470200	32940	F-actin-capping protein subunit alpha-1 OS=Mus musculus OX=10090
Q7TNV0	6813600	2699800	5267500	4092700	43159	Protein DEK OS=Mus musculus OX=10090
P67984	8010600	10288000	7114600	13598000	14759	60S ribosomal protein L22 OS=Mus musculus OX=10090 GN=Rpl22
Q9Z2T6	1075100				55759	Keratin type II cuticular Hb5 OS=Mus musculus OX=10090 GN=Krt85
Q0VBK2	588800	1107700			50661	Keratin type II cytoskeletal 80 OS=Mus musculus OX=10090
Q9D1G1		885660		672240	22187	Ras-related protein Rab-1B OS=Mus musculus OX=10090 GN=Rab1b
Q810V0	377890	2137500	1741200	5422400	78735	U3 small nucleolar ribonucleoprotein protein MPP10 OS=Mus musculus OX=10090
Q8R3H7	2127900	1960300	1098300	2572900	41813	Heparan sulfate 2-O-sulfotransferase 1 OS=Mus musculus

A2BH40	542990	1221800	300010	1784700	242089	AT-rich interactive domain-containing protein 1A OS=Mus
Q91ZW3	1049300	878480	863590	1511900	121627	SWI/SNF-related matrix-associated actin-dependent regulator of chromatin subfamily A member 5 OS=Mus
P84084	1418400	1951000	1143400	1716600	20530	ADP-ribosylation factor 5 OS=Mus musculus OX=10090 GN=Arf5
P61205	1418400	1951000	1143400	1716600	20601	ADP-ribosylation factor 3 OS=Mus musculus OX=10090 GN=Arf3
P84078	1418400	1951000	1143400	1716600	20697	ADP-ribosylation factor 1 OS=Mus musculus OX=10090 GN=Arf1
Q99JX7	1090600	1310700	1115000	446950	70300	Nuclear RNA export factor 1 OS=Mus musculus OX=10090
P97379	584520	847620	704370	1156200	54088	Ras GTPase-activating protein-binding protein 2 OS=Mus musculus
O70194	393240	497780	553340	314340	63989	Eukaryotic translation initiation factor 3 subunit D OS=Mus musculus
Q6GQT1	212410		269140		164352	Alpha-2-macroglobulin-P OS=Mus musculus OX=10090 GN=A2m
P61222	1629900	1558600	1026800	1483900	67314	ATP-binding cassette sub-family E member 1 OS=Mus musculus
Q9CY58	6415100	3325000	2233700	2478900	44714	Plasminogen activator inhibitor 1 RNA-binding protein OS=Mus
P13542	688000	639350	51674	785790	222706	Myosin-8 OS=Mus musculus OX=10090
Q5SX39	688000	639350	51674	785790	222857	Myosin-4 OS=Mus musculus OX=10090
G3UW82	688000	639350	51674	785790	223217	MCG140437 isoform CRA_d OS=Mus musculus OX=10090
Q5SX40	688000	639350	51674	785790	223340	Myosin-1 OS=Mus musculus OX=10090
P13541	688000	639350	51674	785790	223789	Myosin-3 OS=Mus musculus OX=10090
O88569	1146200	1745100	1266900	2526600	37403	Heterogeneous nuclear ribonucleoproteins A2/B1 OS=Mus musculus OX=10090
P97820	1307300	2790800	897840	948280	140602	Mitogen-activated protein kinase kinase kinase kinase 4 OS=Mus
Q8BU30	1647200	2983100	807270	1843300	144270	Isoleucine--tRNA ligase cytoplasmic OS=Mus musculus OX=10090
P05064	174160	3659400			39356	Fructose-bisphosphate aldolase A OS=Mus musculus OX=10090
O08573		2350100	1118300	7973000	40036	Galectin-9 OS=Mus musculus OX=10090
Q9Z320	0			589140	49105	Keratin type I cytoskeletal 27 OS=Mus musculus OX=10090

Q9QZE5	668740	1270400	304090	1087800	97513	Coatomer subunit gamma-1 OS=Mus musculus OX=10090
Q6P1F6	319940	1393400	405520	1406400	51692	Serine/threonine-protein phosphatase 2A 55 kDa regulatory subunit B alpha isoform OS=Mus
Q9CPP0	1702100	2417300	1324600	2026500	19023	Nucleoplasmin-3 OS=Mus musculus
O54781	925710	803880	333370	857600	76757	SRSF protein kinase 2 OS=Mus musculus OX=10090 GN=Srpk2
Q99LE6	1422100	3129000	180910	2200100	71782	ATP-binding cassette sub-family F member 2 OS=Mus musculus
P40630	1629400	3296900	2012500	3867800	27988	Transcription factor A mitochondrial OS=Mus musculus OX=10090
P09803	1631200	1021100	750830	1560300	98256	Cadherin-1 OS=Mus musculus OX=10090
Q61301	658050	852270		716480	105286	Catenin alpha-2 OS=Mus musculus OX=10090
Q61581	1385900	1255200	0		28969	Insulin-like growth factor-binding protein 7 OS=Mus musculus
P35922	95761	241630	112000	472870	68989	Synaptic functional regulator FMR1 OS=Mus musculus OX=10090
P80318	876600	2482600	716800	2181100	60630	T-complex protein 1 subunit gamma OS=Mus musculus OX=10090
P00688	1252800	2954300	1808300	854620	57318	Pancreatic alpha-amylase OS=Mus musculus OX=10090 GN=Amy2
P80315	404060	1194300		426060	58066	T-complex protein 1 subunit delta OS=Mus musculus OX=10090
Q3UTJ2	1752100	2745700		829760	132349	Sorbin and SH3 domain-containing protein 2 OS=Mus musculus
Q7TMB8	1282100	2082000	759430	1477600	145241	Cytoplasmic FMR1-interacting protein 1 OS=Mus musculus
Q8BH04	236070	898700	0	529430	70528	Phosphoenolpyruvate carboxykinase [GTP] mitochondrial OS=Mus musculus OX=10090
O54734	1241600	1113800	125880	284430	49028	Dolichyl-diphosphooligosaccharide--protein glycosyltransferase 48
Q9QYR6			0		300139	Microtubule-associated protein 1A OS=Mus musculus OX=10090
P54823	1846400	1878800	746900	2013100	54192	Probable ATP-dependent RNA helicase DDX6 OS=Mus musculus
Q63918	1501300	2813900	1068100	2099400	46764	Caveolae-associated protein 2 OS=Mus musculus OX=10090
P67778		653250			29820	Prohibitin OS=Mus musculus OX=10090
Q9CWX9	1778400	1190400	204970	619770	50639	Probable ATP-dependent RNA helicase DDX47 OS=Mus musculus

Q9WVA4		2013200		190450	22395	Transgelin-2 OS=Mus musculus OX=10090
Q9D312	344480	333240		287410	49034	Keratin type I cytoskeletal 20 OS=Mus musculus OX=10090
Q9JKC8	1740700	2386700	1039200	1693700	46936	AP-3 complex subunit mu-1 OS=Mus musculus OX=10090 GN=Ap3m1
P70372	1431000	1043700	791550	1393400	36169	ELAV-like protein 1 OS=Mus musculus OX=10090 GN=Elavl1
Q08093	997400	3334700	4377900	3257600	33156	Calponin-2 OS=Mus musculus OX=10090
Q9JJF3	972670	463210	317810	2238900	67557	Ribosomal oxygenase 1 OS=Mus musculus OX=10090 GN=Riox1
Q497I4			4121000		50530	Keratin type I cuticular Ha5 OS=Mus musculus OX=10090 GN=Krt35
E9Q264	3031800	2604900	2781500	1833000	221844	Myosin heavy chain 15 OS=Mus musculus OX=10090 GN=Myh15
P08113	1921300	2976100	0	597610	92476	Endoplasmin OS=Mus musculus OX=10090
P63037	3111700	653400	0	2174900	44868	DnaJ homolog subfamily A member 1 OS=Mus musculus OX=10090
A6BLY7		29033	0		50346	Keratin type I cytoskeletal 28 OS=Mus musculus OX=10090
Q8BK67	270260	624480	170700	158300	55983	Protein RCC2 OS=Mus musculus OX=10090
P46935	568030	912850	303160	532850	102706	E3 ubiquitin-protein ligase NEDD4 OS=Mus musculus OX=10090
Q9DCE5	667830	445910	1687100	1165100	42116	p21-activated protein kinase-interacting protein 1 OS=Mus musculus
Q9QY06	1008100	1967000	681650	1381500	238832	Unconventional myosin-IXb OS=Mus musculus OX=10090 GN=Myo9b
Q8CGK3	278490	707750	218720	213320	105843	Lon protease homolog mitochondrial OS=Mus musculus OX=10090
G5E870	195540	495920	215310	329000	224126	E3 ubiquitin-protein ligase TRIP12 OS=Mus musculus OX=10090
P60335	1759100	3076400	1258900	2251200	37498	Poly(rC)-binding protein 1 OS=Mus musculus OX=10090 GN=Pcbp1
Q6A065	785990	430740	152090	692430	175049	Centrosomal protein of 170 kDa OS=Mus musculus OX=10090
Q61495	3908300	1308600	1220200	1630400	114597	Desmoglein-1-alpha OS=Mus musculus OX=10090 GN=Dsg1a
Q7TSF1	3908300	1308600	1220200	1630400	114454	Desmoglein-1-beta OS=Mus musculus
O54825	1174100	809750	419610	894520	49784	Bystin OS=Mus musculus OX=10090
Q99LH1	196200	0	177300	524150	83346	Nucleolar GTP-binding protein 2 OS=Mus musculus OX=10090
B1AZI6	751600	841590	431910	933330	182772	THO complex subunit 2 OS=Mus musculus OX=10090 GN=Thoc2

Q920B9	1985900	4263600	116320	3433500	119825	FACT complex subunit SPT16 OS=Mus musculus OX=10090
Q8CI43	2417500	2291300		2369100	22749	Myosin light chain 6B OS=Mus musculus OX=10090 GN=Myl6b
P07146	928980	2010300		1415900	26204	Anionic trypsin-2 OS=Mus musculus
Q91Z67	181740	1504500	923030	1582400	120798	SLIT-ROBO Rho GTPase-activating protein 2 OS=Mus
Q8VE37	1398100	1748700	645140	244860	44931	Regulator of chromosome condensation OS=Mus
Q99JB8	610940	1487600			48585	Protein kinase C and casein kinase II substrate protein 3 OS=Mus
Q3UEB3	10648000	3577500	71221	4102500	60249	Poly(U)-binding-splicing factor PUF60 OS=Mus musculus OX=10090
P51859	4313300	2888700	1748400	607990	26269	Hepatoma-derived growth factor OS=Mus musculus OX=10090
O54791	2019000	1107200	1306600	780280	16955	Transcription factor MafF OS=Mus musculus OX=10090 GN=Maff
Q9EQ06	214310				32881	Estradiol 17-beta-dehydrogenase 11 OS=Mus musculus
P49718	859690	870720		858610	82407	DNA replication licensing factor MCM5 OS=Mus musculus
Q8BWY3	2471400	2509200	1004100	2080800	49031	Eukaryotic peptide chain release factor subunit 1 OS=Mus musculus
Q8C8U0	1825700	674790	365900	694720	108540	Liprin-beta-1 OS=Mus musculus OX=10090
Q62318	310840	926480	324450	2933500	88847	Transcription intermediary factor 1-beta OS=Mus musculus
Q08288	2713400	2687700	411800	3051000	43736	Cell growth-regulating nucleolar protein OS=Mus musculus
P42932	394180	2260300	79665	0	59556	T-complex protein 1 subunit theta OS=Mus musculus OX=10090
P10833	683100	1213900		1049600	23764	Ras-related protein R-Ras OS=Mus musculus OX=10090 GN=Rras
P56959	1509100	3085400	890460	1463000	52673	RNA-binding protein FUS OS=Mus musculus OX=10090 GN=Fus
Q8VI75	806280	961340	642240	515280	119275	Importin-4 OS=Mus musculus OX=10090
Q6ZPF4	931510	1519300	296550	796880	117169	Formin-like protein 3 OS=Mus musculus OX=10090 GN=Fmnl3
A2APV2	931510	1519300	296550	796880	123101	Formin-like protein 2 OS=Mus musculus OX=10090 GN=Fmnl2
P02469	563440	922110	166960	712740	197089	Laminin subunit beta-1 OS=Mus musculus OX=10090 GN=Lamb1
Q8R409	537830	1427400	657310	1144000	40243	Protein HEXIM1 OS=Mus musculus

G3UZK1	1159300	1028400	449820	793490	16000	Guanine nucleotide-binding protein subunit gamma OS=Mus musculus OX=10090
A2ALW5	1159300	1028400	449820	793490	41937	DnaJ homolog subfamily C member 25 OS=Mus musculus OX=10090
Q8BMC4		464700		644630	70047	Nucleolar protein 9 OS=Mus musculus
Q91VE6		2858400		2218900	36265	MKI67 FHA domain-interacting nucleolar phosphoprotein OS=Mus musculus OX=10090
A2A4X6	216050	0	2002700	2212800	19130	MCG21910 OS=Mus musculus OX=10090 GN=Gm12355 PE=4
P84104	216050	0	2002700	2212800	19330	Serine/arginine-rich splicing factor 3 OS=Mus musculus OX=10090
P10107		307730		210860	38734	Annexin A1 OS=Mus musculus OX=10090
Q9EPL8	189040	494390		192500	119486	Importin-7 OS=Mus musculus OX=10090
P26231		1365100			100106	Catenin alpha-1 OS=Mus musculus OX=10090
E9PYD1	292180	216540	620680	143170	37272	Family with sequence similarity 98 member C OS=Mus musculus
O88685		882700			49549	26S proteasome regulatory subunit 6A OS=Mus musculus
Q4FZF3	146450	176320		125790	54094	Probable ATP-dependent RNA helicase DDX49 OS=Mus musculus
Q921Y2	1759400	1902900	1100900	2863600	21777	U3 small nucleolar ribonucleoprotein protein IMP3 OS=Mus musculus
Q922R8	284140	1397100	261240	272040	48100	Protein disulfide-isomerase A6 OS=Mus musculus OX=10090
E9Q616	3156900	3720600	996150	3188000	604264	AHNAK nucleoprotein (desmoyokin) OS=Mus musculus OX=10090
Q8K2B3	267840	543830	183030	423460	72585	Succinate dehydrogenase [ubiquinone] flavoprotein subunit mitochondrial OS=Mus musculus
P60122	287550	880650	381690	692940	50214	RuvB-like 1 OS=Mus musculus OX=10090
Q9CQI7	1019500	1316700	511660	934050	25323	U2 small nuclear ribonucleoprotein B" OS=Mus musculus
E9Q7G0		696190	484070	461640	235628	Nuclear mitotic apparatus protein 1 OS=Mus musculus OX=10090
P17095	10372000	2749900	1991800	3502100	11614	High mobility group protein HMG-I/HMG-Y OS=Mus musculus
Q60960		483670	470070	353190	60183	Importin subunit alpha-5 OS=Mus musculus OX=10090 GN=Kpna1
Q9D945	1754200			2547100	15391	Protein LLP homolog OS=Mus musculus OX=10090 GN=Llph

O08810		1390600		1435300	109361	116 kDa U5 small nuclear ribonucleoprotein component OS=Mus musculus OX=10090
O35218	271140	531390	277890	543490	88383	Cleavage and polyadenylation specificity factor subunit 2 OS=Mus musculus
Q08943	62116	1127500	62637	912750	80860	FACT complex subunit SSRP1 OS=Mus musculus OX=10090
P28656	2774600	3268000		2447600	45345	Nucleosome assembly protein 1-like 1 OS=Mus musculus OX=10090
Q99NB9	0	1814700	0	0	145816	Splicing factor 3B subunit 1 OS=Mus musculus OX=10090
Q01853	141790	2357500	379350		89322	Transitional endoplasmic reticulum ATPase OS=Mus musculus
Q9JHU4	826350	235950	359760	817480	532050	Cytoplasmic dynein 1 heavy chain 1 OS=Mus musculus OX=10090
O35231	0	130400	132020	0	92556	Kinesin-like protein KIFC3 OS=Mus musculus OX=10090
Q8VDJ3	1530900	2434400	902340	1234800	141742	Vigilin OS=Mus musculus OX=10090
P31230	666400	1430600	160710	835800	33997	Aminoacyl tRNA synthase complex-interacting multifunctional protein 1
Q6PDM2	1669200	1365100	1036000	1295500	27745	Serine/arginine-rich splicing factor 1 OS=Mus musculus OX=10090
P24788	352900		109630	335350	91514	Cyclin-dependent kinase 11B OS=Mus musculus OX=10090 GN=Cdk11b
Q8R361	1157600	1219100		849510	69553	Rab11 family-interacting protein 5 OS=Mus musculus OX=10090
Q8BX10	1680300	830830	875330	1476800	31994	Serine/threonine-protein phosphatase PGAM5 mitochondrial OS=Mus musculus OX=10090
P61750		0		0	20397	ADP-ribosylation factor 4 OS=Mus musculus OX=10090 GN=Arf4
Q9DBB4	131950	563750	176080		101284	N-alpha-acetyltransferase 16 NatA auxiliary subunit OS=Mus
Q9QX47	240740	270320	294350	299620	265649	Protein SON OS=Mus musculus OX=10090
O54988	1891800	2398800	987460	1640500	141457	STE20-like serine/threonine-protein kinase OS=Mus
Q8CB77	3201200	2333600	1558800	3112400	87161	Elongin-A OS=Mus musculus OX=10090
Q3UJB0	1628700	1724100	1640500	1330800	98199	Splicing factor 3b subunit 2 OS=Mus musculus OX=10090
P49312	894670	1331300	1237100	1399100	34196	Heterogeneous nuclear ribonucleoprotein A1 OS=Mus musculus OX=10090 GN=Hnrnpa1

O89079	887820	1987800	792460	1667800	34567	Coatomer subunit epsilon OS=Mus musculus OX=10090 GN=Cope
Q9DBJ3	564200	324090	660250	281180	57188	Brain-specific angiogenesis inhibitor 1-associated protein 2-like protein 1 OS=Mus
E9Q1F5	907000	827060		1029800	202701	Myosin VC OS=Mus musculus OX=10090
P46662		316440	242950	2975900	69776	Merlin OS=Mus musculus OX=10090
Q8R3N1	1390200	1520400	443240	1764400	98769	Nucleolar protein 14 OS=Mus musculus OX=10090 GN=Nop14
Q8K310	1007100	1791200		948970	94630	Matrin-3 OS=Mus musculus OX=10090
P70168	519850	595010		515940	97184	Importin subunit beta-1 OS=Mus musculus OX=10090 GN=Kpnb1
P08905	2139000	3002200	769450	1525400	16689	Lysozyme C-2 OS=Mus musculus OX=10090
P17897	2139000	3002200	769450	1525400	16794	Lysozyme C-1 OS=Mus musculus OX=10090
Q05895	0				104119	Thrombospondin-3 OS=Mus musculus
Q64285	880200	686910		822480	65813	Bile salt-activated lipase OS=Mus musculus OX=10090 GN=Cel
A0A2I3BRL8			1175100		42179	Predicted gene 7324 OS=Mus musculus OX=10090 GN=Gm7324
Q91VM5			1175100		42162	RNA binding motif protein X-linked-like-1 OS=Mus musculus
Q9WV02			1175100		42301	RNA-binding motif protein X chromosome OS=Mus musculus
Q9D937	1144400			2012000	14099	Uncharacterized protein C11orf98 homolog OS=Mus musculus
A0A087WRC	1144400			2012000	11508	Predicted gene 49416 OS=Mus musculus OX=10090
P51150	183920	1670700			23490	Ras-related protein Rab-7a OS=Mus musculus OX=10090 GN=Rab7a
Q8BH59	281790	304790		206430	74570	Calcium-binding mitochondrial carrier protein Aralar1 OS=Mus
Q8BGT7	2601200	2386100	2068700	2470900	26753	Survival of motor neuron-related-splicing factor 30 OS=Mus
Q924W7	984910	1126400	745540	1283900	126861	Suppression of tumorigenicity 5 protein OS=Mus musculus
P13864	249170	696930	201510	676640	183188	DNA (cytosine-5)-methyltransferase 1 OS=Mus musculus
Q60865		792510	2308200	579250	78169	Caprin-1 OS=Mus musculus OX=10090
Q61092		475580	47328	420560	130161	Laminin subunit gamma-2 OS=Mus musculus OX=10090 GN=Lamc2
Q9R0T7		67859000	1348700	52816000	26274	MCG15085 OS=Mus musculus OX=10090

Q9QUK9		67859000	1348700	52816000	26277	MCG15083 OS=Mus musculus OX=10090
Q8K2C9	169600	299920	125430	666960	43131	Very-long-chain (3R)-3-hydroxyacyl-CoA dehydratase 3 OS=Mus
Q9R190	1370000	1055800	436990	1222900	75030	Metastasis-associated protein MTA2 OS=Mus musculus OX=10090
Q91W50	147950	223340			88791	Cold shock domain-containing protein E1 OS=Mus musculus
Q9Z1Q5		3062800			27013	Chloride intracellular channel protein 1 OS=Mus musculus
O09118			480250	117360	67810	Netrin-1 OS=Mus musculus OX=10090
Q61194	1329400	1605900	90089	1189300	190757	Phosphatidylinositol 4-phosphate 3-kinase C2 domain-containing subunit alpha OS=Mus
Q3UFY8	0	266060	137930	1254800	48386	tRNA methyltransferase 10 homolog C OS=Mus musculus OX=10090
Q8CH25		144700		826380	116920	SAFB-like transcription modulator OS=Mus musculus OX=10090
Q8BGA5	992830	1171600	1121300	1308200	43538	KRR1 small subunit processome component homolog OS=Mus musculus OX=10090
P70279	1159300	1609000	1147000	1722500	41235	Surfeit locus protein 6 OS=Mus musculus OX=10090 GN=Surf6
P09411		5565700			44550	Phosphoglycerate kinase 1 OS=Mus musculus OX=10090 GN=Pgk1
Q62266	1716400	5518000	411680	411720	15765	Cornifin-A OS=Mus musculus OX=10090
Q9CY27		0	104890	669730	36090	Very-long-chain enoyl-CoA reductase OS=Mus musculus OX=10090
Q8CBE3	50261	280690	186000	249680	55046	WD repeat-containing protein 37 OS=Mus musculus OX=10090
Q9QXY1			230730	221640	99324	Tight junction protein ZO-3 OS=Mus musculus OX=10090 GN=Tjp3
Q9ERG0	213720	140640		0	84060	LIM domain and actin-binding protein 1 OS=Mus musculus
Q99KI0		713380			85464	Aconitate hydratase mitochondrial OS=Mus musculus OX=10090
P97376	1770600	1346700	858760	1612400	29127	Protein FRG1 OS=Mus musculus OX=10090
Q6PGH1	453860	229170	197600	0	17000	Protein BUD31 homolog OS=Mus musculus OX=10090 GN=Bud31
O09106	0	638270	278440	962610	55075	Histone deacetylase 1 OS=Mus musculus OX=10090 GN=Hdac1
Q6NZJ6	572690	158690		0	176076	Eukaryotic translation initiation factor 4 gamma 1 OS=Mus musculus

P59999	718340	1240700	102610	273570	19667	Actin-related protein 2/3 complex subunit 4 OS=Mus musculus
Q9CY50	697150	801450	387080	1020200	32065	Translocon-associated protein subunit alpha OS=Mus musculus
P53569	678400	857480	173070	964020	120262	CCAAT/enhancer-binding protein zeta OS=Mus musculus
Q9CYH6	906140	878960	689950	828780	41552	Ribosome biogenesis regulatory protein homolog OS=Mus
Q8VI84		100520	382140	0	93211	Nucleolar complex protein 3 homolog OS=Mus musculus
Q9DBD5	0	133910		113170	118069	Proline- glutamic acid- and leucine-rich protein 1 OS=Mus musculus
Q8BSY0	349750	650660	109520		83042	Aspartyl/asparaginyl beta-hydroxylase OS=Mus musculus
P63038		581430	278220	341200	60956	60 kDa heat shock protein mitochondrial OS=Mus musculus
Q60668	142740	237570	1013000		38354	Heterogeneous nuclear ribonucleoprotein D0 OS=Mus musculus OX=10090 GN=Hnrnpd
Q9Z130	142740	237570	0		33559	Heterogeneous nuclear ribonucleoprotein D-like OS=Mus musculus OX=10090 GN=Hnrnpdl
A2AMD0		143670		81999	28466	Predicted gene 12666 (Fragment) OS=Mus musculus OX=10090
Q05CL8		143670		81999	64802	La-related protein 7 OS=Mus musculus
P14824		261480			75885	Annexin A6 OS=Mus musculus OX=10090
Q99MJ9		147190	104490	149770	82176	ATP-dependent RNA helicase DDX50 OS=Mus musculus
Q61545	499380	817060			68462	RNA-binding protein EWS OS=Mus musculus OX=10090 GN=Ewsr1
Q61771				924280	85288	Kinesin-like protein KIF3B OS=Mus musculus OX=10090
O35066				924280	89973	Kinesin-like protein KIF3C OS=Mus musculus OX=10090
P97855	0	0	39767		51829	Ras GTPase-activating protein-binding protein 1 OS=Mus musculus
B2RS76		601230		109080	47574	Carboxypeptidase B1 (Tissue) isoform CRA_b OS=Mus musculus
Q8VEH6		241130	393870	504080	43772	COBW domain-containing protein 1 OS=Mus musculus
P56960	0	929880	420160		100942	Exosome component 10 OS=Mus musculus OX=10090 GN=Exosc10
Q8CHW4	1470900	1066700		992540	80086	Translation initiation factor eIF-2B subunit epsilon OS=Mus

Q9D154		957210	441680	165790	42575	Leukocyte elastase inhibitor A OS=Mus musculus OX=10090
P63158	620820		324720	401510	24894	High mobility group protein B1 OS=Mus musculus OX=10090
Q5BLK4	375680	466600		0	169102	Terminal uridylyltransferase 7 OS=Mus musculus
Q99JR5	836320	331420		239590	52665	Tubulointerstitial nephritis antigen-like OS=Mus musculus
Q505F5		581090		265610	63590	Leucine-rich repeat-containing protein 47 OS=Mus musculus
Q80XC3		120800		192180	93574	USP6 N-terminal-like protein OS=Mus musculus OX=10090
Q9WUM4	1016700	1384900	524150	1409700	53121	Coronin-1C OS=Mus musculus OX=10090
Q9EQI8	448940	1013900		1776000	32132	39S ribosomal protein L46 mitochondrial OS=Mus musculus
Q9CR02	1530400	1581300	1123700	2182600	25782	Translation machinery-associated protein 16 OS=Mus musculus
Q9JKX4	582490	1244000	420630	1691600	59482	Protein AATF OS=Mus musculus OX=10090
Q61838				0	165852	Pregnancy zone protein OS=Mus musculus OX=10090 GN=Pzp
Q9DC71		274400	216250	347740	29464	28S ribosomal protein S15 mitochondrial OS=Mus musculus
Q9JMB0	511230	390050	341400	461370	41763	G kinase-anchoring protein 1 OS=Mus musculus OX=10090
Q9D883	626220	997580		1126800	27815	Splicing factor U2AF 35 kDa subunit OS=Mus musculus OX=10090
Q8BGJ9	626220	997580		1126800	25836	Splicing factor U2AF 26 kDa subunit OS=Mus musculus OX=10090
P56183	904270	0		1054700	54777	Ribosomal RNA processing protein 1 homolog A OS=Mus
Q8C4J7	0			91881	88266	Transducin beta-like protein 3 OS=Mus musculus OX=10090
Q8JZU2	1172000	1433300	597740	1275400	33932	Tricarboxylate transport protein mitochondrial OS=Mus musculus
Q9WVR4	862980		0		73743	Fragile X mental retardation syndrome-related protein 2
Q80X98	168750	153850	113550	196030	140622	DEAH (Asp-Glu-Ala-His) box polypeptide 38 OS=Mus musculus
Q922H9	1229100	623560	655520	740610	35608	Zinc finger protein 330 OS=Mus musculus OX=10090 GN=Znf330
P23116	594860	1743100	260090	668460	161936	Eukaryotic translation initiation factor 3 subunit A OS=Mus musculus

Q9Z110		0			87266	Delta-1-pyrroline-5-carboxylate synthase OS=Mus musculus
O88967		1242200			80028	ATP-dependent zinc metalloprotease YME1L1 OS=Mus
Q8R081		673250	721500	702340	63964	Heterogeneous nuclear ribonucleoprotein L OS=Mus musculus
Q91VH2	267590	416930	306180	358720	66546	Sorting nexin-9 OS=Mus musculus OX=10090
Q8R1B4	196610	915160		0	105531	Eukaryotic translation initiation factor 3 subunit C OS=Mus musculus
Q5RJG1	259500			466600	80077	Nucleolar protein 10 OS=Mus musculus OX=10090 GN=Nol10
Q921I9	1308200	1572200	846960	1324400	26250	Exosome complex component RRP41 OS=Mus musculus
P54071	0	1077200			50906	Isocitrate dehydrogenase [NADP] mitochondrial OS=Mus musculus
O08992	191290	282920	88740	386890	32379	Syntenin-1 OS=Mus musculus OX=10090
P09602			2204100	1452500	9423	Non-histone chromosomal protein HMG-17 OS=Mus
Q9DBJ1	56777	416670			28832	Phosphoglycerate mutase 1 OS=Mus musculus OX=10090 GN=Pgam1
Q9Z2Q5	264170	2652300	1924200	2952700	24301	39S ribosomal protein L40 mitochondrial OS=Mus musculus
Q9QXB9	338430	612760	323090	580310	40718	Developmentally-regulated GTP-binding protein 2 OS=Mus
P54103	121640	178830	124700	159280	71722	DnaJ homolog subfamily C member 2 OS=Mus musculus OX=10090
Q61753		1236600	545090	0	56586	D-3-phosphoglycerate dehydrogenase OS=Mus musculus OX=10090
Q6A0A9	204480	492740	292600	206080	121646	Constitutive coactivator of PPAR-gamma-like protein 1 OS=Mus musculus OX=10090
Q8BML9	0	0		381640	87677	Glutamine--tRNA ligase OS=Mus musculus OX=10090 GN=Qars
Q8K1E0	239410	265930	123110		39713	Syntaxin-5 OS=Mus musculus OX=10090
Q80XP8	856850	816030	479880	975430	38556	Protein FAM76B OS=Mus musculus
Q91YK2		561970		1440800	80582	Ribosomal RNA processing protein 1 homolog B OS=Mus
Q923D5	1198500	0			69875	WW domain-binding protein 11 OS=Mus musculus OX=10090
Q8BMJ2		1295900			134192	Leucine--tRNA ligase cytoplasmic OS=Mus musculus OX=10090
Q8C092	672260			363700	87045	Transcription initiation factor TFIID subunit 5 OS=Mus musculus

Q61166		970140			30016	Microtubule-associated protein RP/EB family member 1 OS=Mus
Q9DB96	1479100			139770	35659	Neuroguidin OS=Mus musculus OX=10090
Q61792	1979600	1845900	1107200	1828900	29994	LIM and SH3 domain protein 1 OS=Mus musculus OX=10090
Q8BUN5	1210400		462430		48081	Mothers against decapentaplegic homolog 3 OS=Mus musculus
O35613			354280	859020	81489	Death domain-associated protein 6 OS=Mus musculus OX=10090
P48678	742480	352290		0	74238	Prelamin-A/C OS=Mus musculus OX=10090
Q8BK35			197710	231860	55793	Ribosome biogenesis protein NOP53 OS=Mus musculus OX=10090
Q91YR7		288650		0	106722	Pre-mRNA-processing factor 6 OS=Mus musculus OX=10090
P26638	243480	463250	147880	115030	58389	Serine--tRNA ligase cytoplasmic OS=Mus musculus OX=10090
Q9Z0H3		3735700	2831000	765100	44141	SWI/SNF-related matrix-associated actin-dependent regulator of chromatin subfamily B member 1 OS=Mus
Q9DCH4	246920	231620		245700	37984	Eukaryotic translation initiation factor 3 subunit F OS=Mus musculus
Q9D0D4	473360	240880		223410	35274	Probable dimethyladenosine transferase OS=Mus
Q99LC3	117480	224320		175100	40603	NADH dehydrogenase [ubiquinone] 1 alpha subcomplex subunit 10 mitochondrial OS=Mus
Q9JKR6	289140	1027500	172850	283050	111181	Hypoxia up-regulated protein 1 OS=Mus musculus OX=10090
Q9D1R9	1795100	3633100	1882200	2193900	13293	60S ribosomal protein L34 OS=Mus musculus OX=10090 GN=Rpl34
P23927		412060		989570	20069	Alpha-crystallin B chain OS=Mus musculus OX=10090 GN=Cryab
Q68FL6	1692000	1862900	1047900	0	101431	Methionine--tRNA ligase cytoplasmic OS=Mus musculus OX=10090
A2AJI0	941850	1694900	782980	718760	93276	MAP7 domain-containing protein 1 OS=Mus musculus
Q8R5C5		315610		445140	42281	Beta-centractin OS=Mus musculus OX=10090
P61164		315610		445140	42614	Alpha-centractin OS=Mus musculus
Q8CFI7	177120	185180		1199100	133910	DNA-directed RNA polymerase II subunit RPB2 OS=Mus musculus
Q60973				400860	47790	Histone-binding protein RBBP7 OS=Mus musculus OX=10090

Q9WTM5	162570	769380	122940	320180	51113	RuvB-like 2 OS=Mus musculus OX=10090
A8DUK4	832300	1511900	905080	1012800	15748	Beta-globin OS=Mus musculus OX=10090
P02089	832300	1511900	905080	1012800	15878	Hemoglobin subunit beta-2 OS=Mus musculus OX=10090
P02088	832300	1511900	905080	1012800	15840	Hemoglobin subunit beta-1 OS=Mus musculus OX=10090
P02104	832300	1511900	905080	1012800	16137	Hemoglobin subunit epsilon-Y2 OS=Mus musculus OX=10090
Q3TJZ6	841510	1444600	380710		55055	Protein FAM98A OS=Mus musculus
Q05512	135080		757560		86306	Serine/threonine-protein kinase MARK2 OS=Mus musculus OX=10090
Q60760	111750	155690	116820	170670	70585	Growth factor receptor-bound protein 10 OS=Mus musculus
Q3TXS7		64877			105730	26S proteasome non-ATPase regulatory subunit 1 OS=Mus
Q9JM76	91557	212060			20525	Actin-related protein 2/3 complex subunit 3 OS=Mus musculus
Q3THS6	1028200	1129800			43689	S-adenosylmethionine synthase isoform type-2 OS=Mus musculus
P54869		858030			56823	Hydroxymethylglutaryl-CoA synthase mitochondrial OS=Mus
P12382			0	328130	85360	ATP-dependent 6-phosphofructokinase liver type OS=Mus
Q8BU14	591800	2182300	421550	1335700	45581	Translocation protein SEC62 OS=Mus musculus OX=10090
O35658		272030			31013	Complement component 1 Q subcomponent-binding protein mitochondrial OS=Mus
Q9CYX7	1526900	1240000	982390	1241100	31058	RRP15-like protein OS=Mus musculus
P59325	125890	288840	111790	174190	48968	Eukaryotic translation initiation factor 5 OS=Mus musculus
Q9CXY6	276760	1863900	415440	539390	43062	Interleukin enhancer-binding factor 2 OS=Mus musculus OX=10090
Q99KV1	3050200	2131000	1434200	1808600	40555	DnaJ homolog subfamily B member 11 OS=Mus musculus OX=10090
Q9CY21	2081100	1607000	713350	2386400	31587	Probable 18S rRNA (guanine-N(7))-methyltransferase
O54785	607850	566520	346640	370320	72202	LIM domain kinase 2 OS=Mus musculus OX=10090 GN=Limk2
Q8CD10	1144000		727920	1555300	49476	Calcium uptake protein 2 mitochondrial OS=Mus musculus OX=10090
Q4VA53	162750				164418	Sister chromatid cohesion protein PDS5 homolog B OS=Mus

Q62074		1093400		1177600	68204	Protein kinase C iota type OS=Mus musculus OX=10090 GN=Prkci
Q4VBE8	324180	182560	68368	0	47211	WD repeat-containing protein 18 OS=Mus musculus OX=10090
Q09200				0	59212	Beta-1 4 N-acetylgalactosaminyltransferase 1 OS=Mus musculus OX=10090
Q6NS46		196400		105510	207777	Protein RRP5 homolog OS=Mus musculus OX=10090 GN=Pdcd11
P68181	219660	269500			40708	cAMP-dependent protein kinase catalytic subunit beta OS=Mus musculus OX=10090 GN=Prkacb
Q9JIF7	26888			137310	107066	Coatomer subunit beta OS=Mus musculus OX=10090 GN=Copb1
Q9D0D5	187970				49593	General transcription factor IIE subunit 1 OS=Mus musculus
P02468	703180	792440	383050	718880	177298	Laminin subunit gamma-1 OS=Mus musculus OX=10090 GN=Lamc1
P63242	0	611320	0		16832	Eukaryotic translation initiation factor 5A-1 OS=Mus musculus
Q8BG17			378660	767190	25356	Nucleolar protein 12 OS=Mus musculus OX=10090 GN=Nol12
Q9D0M3	596100	921690		277810	35328	Cytochrome c1 heme protein mitochondrial OS=Mus musculus
P70336		240880	113010	251420	160585	Rho-associated protein kinase 2 OS=Mus musculus OX=10090
Q9WU56	2050400	1895700	1403100	1664300	47502	tRNA pseudouridine synthase A OS=Mus musculus OX=10090
P35601	175370	280020		151310	125985	Replication factor C subunit 1 OS=Mus musculus OX=10090
Q6PGF5	261480		99904		145413	BMS1 homolog ribosome assembly protein (Yeast) OS=Mus
P63330			0		35608	Serine/threonine-protein phosphatase 2A catalytic subunit alpha isoform OS=Mus musculus
P62715			0		35575	Serine/threonine-protein phosphatase 2A catalytic subunit beta isoform OS=Mus musculus
P17751	2459900	363310		483750	32192	Triosephosphate isomerase OS=Mus musculus OX=10090
Q91VN6	790510	735520	482230	834800	69820	Probable ATP-dependent RNA helicase DDX41 OS=Mus musculus
P70412		253840		0	68082	CUB and zona pellucida-like domain-containing protein 1 OS=Mus

P62492		270080			24394	Ras-related protein Rab-11A OS=Mus musculus OX=10090 GN=Rab11a
P46638		270080			24489	Ras-related protein Rab-11B OS=Mus musculus OX=10090 GN=Rab11b
P28741	243700	280270			80170	Kinesin-like protein KIF3A OS=Mus musculus OX=10090
Q07235	416290			145200	44207	Glia-derived nexin OS=Mus musculus
Q01730			0	1697500	31550	Ras suppressor protein 1 OS=Mus musculus OX=10090 GN=Rsu1
P42125	158360			222940	32250	Enoyl-CoA delta isomerase 1 mitochondrial OS=Mus
Q9ESL4		134330			91720	Mitogen-activated protein kinase kinase kinase 20 OS=Mus
Q6PAC3	680710			682960	51456	DDB1- and CUL4-associated factor 13 OS=Mus musculus
Q9D0R4	410990	1290300		1309200	61212	Probable ATP-dependent RNA helicase DDX56 OS=Mus musculus
Q8VCX5		838340		926770	54353	Calcium uptake protein 1 mitochondrial OS=Mus musculus OX=10090
Q9CR67	958980	857580	460790	1209400	28031	Transmembrane protein 33 OS=Mus musculus OX=10090 GN=Tmem33
Q99K30		42142			82229	Epidermal growth factor receptor kinase substrate 8-like protein 2 OS=Mus musculus OX=10090
P53994		177700			23548	Ras-related protein Rab-2A OS=Mus musculus OX=10090 GN=Rab2a
P59279		177700			24198	Ras-related protein Rab-2B OS=Mus musculus OX=10090 GN=Rab2b
Q8C854		723960			63295	Myelin expression factor 2 OS=Mus musculus OX=10090 GN=Myef2
P43883		2420500	826850	1367200	46646	Perilipin-2 OS=Mus musculus OX=10090
O35343	599760	606830	480650		57923	Importin subunit alpha-3 OS=Mus musculus OX=10090 GN=Kpna4
Q64331	656310			859880	146409	Unconventional myosin-VI OS=Mus musculus OX=10090 GN=Myo6
Q8JZW4	124300	0	0		65592	Copine-5 OS=Mus musculus OX=10090
Q8BT60	124300	0	0		59585	Copine-3 OS=Mus musculus OX=10090
P59108	124300	0	0		61036	Copine-2 OS=Mus musculus OX=10090
Q1RLL3	124300	0	0		61852	Copine-9 OS=Mus musculus OX=10090
Q8BLR2	124300	0	0		62408	Copine-4 OS=Mus musculus OX=10090
Q9Z140	124300	0	0		61781	Copine-6 OS=Mus musculus OX=10090

Q0VE82	124300	0	0		61891	Copine-7 OS=Mus musculus OX=10090
Q9DC53	124300	0	0		64667	Copine-8 OS=Mus musculus OX=10090
Q921X9		1328900	447760	573080	59267	Protein disulfide-isomerase A5 OS=Mus musculus OX=10090
Q8K4L0	110220				97748	ATP-dependent RNA helicase DDX54 OS=Mus musculus
E9Q8D0	331310			289080	61735	DnaJ homolog subfamily C member 21 OS=Mus musculus OX=10090
Q61584			320490	472570	76222	Fragile X mental retardation syndrome-related protein 1
Q62186		767070			18937	Translocon-associated protein subunit delta OS=Mus musculus
Q9Z0H1		109790	237980	896140	69048	WD repeat-containing protein 46 OS=Mus musculus OX=10090
Q920Q6		0			36939	RNA-binding protein Musashi homolog 2 OS=Mus musculus
Q5SSK3	152790			277580	41874	Transcription elongation factor mitochondrial OS=Mus musculus
G3UYQ4	931120	996950	699690	702250	219532	Adenylate kinase 9 OS=Mus musculus
A0A1B0GRV'	931120	996950	699690	702250	24262	Predicted gene 17949 OS=Mus musculus OX=10090
Q9WTP6	931120	996950	699690	702250	26469	Adenylate kinase 2 mitochondrial OS=Mus musculus OX=10090
Q8CGC6	274070	263880		176530	84214	RNA-binding protein 28 OS=Mus musculus OX=10090 GN=Rbm28
Q9DBN5		0			94526	Lon protease homolog 2 peroxisomal OS=Mus musculus OX=10090
P70188		784760			91291	Kinesin-associated protein 3 OS=Mus musculus OX=10090
Q9CZX0	275880	376660	196570	280410	62385	Elongator complex protein 3 OS=Mus musculus OX=10090
P58468		207390			24814	Protein FAM207A OS=Mus musculus
Q8VDN2		1065300			112982	Sodium/potassium-transporting ATPase subunit alpha-1 OS=Mus
Q6PIE5		1065300			112217	Sodium/potassium-transporting ATPase subunit alpha-2 OS=Mus
Q6PIC6		1065300			111691	Sodium/potassium-transporting ATPase subunit alpha-3 OS=Mus
Q61316		603270			94133	Heat shock 70 kDa protein 4 OS=Mus musculus OX=10090
P37040	290420			432850	77044	NADPH--cytochrome P450 reductase OS=Mus musculus OX=10090

Q8VEJ9	0	346260		291140	48907	Vacuolar protein sorting-associated protein 4A OS=Mus musculus
P46467	0	346260		291140	49419	Vacuolar protein sorting-associated protein 4B OS=Mus musculus
Q8BPY9	0	346260		291140	74851	Fidgetin-like protein 1 OS=Mus musculus OX=10090 GN=Fignl1
P08775		458200		582010	217174	DNA-directed RNA polymerase II subunit RPB1 OS=Mus musculus
Q8BJL1	147930			0	82688	F-box only protein 30 OS=Mus musculus OX=10090 GN=Fbxo30
P25206	249730	235180	166110	217980	91546	DNA replication licensing factor MCM3 OS=Mus musculus
Q9CRB2	1365400		893190	1672300	17247	H/ACA ribonucleoprotein complex subunit 2
Q9CZD3		416110	182530		81878	Glycine--tRNA ligase OS=Mus musculus OX=10090 GN=Gars
Q8BXK8		814890		824240	94411	Arf-GAP with GTPase ANK repeat and PH domain-containing protein 1 OS=Mus
Q8VHH5		814890		824240	97965	Arf-GAP with GTPase ANK repeat and PH domain-containing protein 3 OS=Mus
P08752	546580				40489	Guanine nucleotide-binding protein G(i) subunit alpha-2 OS=Mus musculus OX=10090
B2RSH2	546580				40361	Guanine nucleotide-binding protein G(i) subunit alpha-1 OS=Mus musculus OX=10090
Q3V3I2	546580				40316	Guanine nucleotide-binding protein G(t) subunit alpha-3 OS=Mus musculus OX=10090
Q6R0H7	546580				121505	Guanine nucleotide-binding protein G(s) subunit alpha isoforms XLas OS=Mus musculus
P18872	546580				40085	Guanine nucleotide-binding protein G(o) subunit alpha OS=Mus
P50149	546580				40118	Guanine nucleotide-binding protein G(t) subunit alpha-2 OS=Mus musculus OX=10090
P27601	546580				44055	Guanine nucleotide-binding protein subunit alpha-13 OS=Mus
P27600	546580				44095	Guanine nucleotide-binding protein subunit alpha-12 OS=Mus
Q9DC51	546580				40538	Guanine nucleotide-binding protein G(i) subunit alpha OS=Mus

Q8CGK7	546580				44309	Guanine nucleotide-binding protein G(olf) subunit alpha OS=Mus musculus OX=10090
P20612	546580				39967	Guanine nucleotide-binding protein G(t) subunit alpha-1 OS=Mus musculus OX=10090
Q9Z2Q2	1573300		745100		53846	Lysine-rich nucleolar protein 1 OS=Mus musculus OX=10090
Q8BGX0		80212			63931	E3 ubiquitin-protein ligase TRIM23 OS=Mus musculus OX=10090
P62331		80212			20082	ADP-ribosylation factor 6 OS=Mus musculus OX=10090 GN=Arf6
Q99MN1	44796	168300	75029	0	67840	Lysine--tRNA ligase OS=Mus musculus OX=10090 GN=Kars
Q6P8K8	0			233870	47339	Carboxypeptidase A4 OS=Mus musculus OX=10090 GN=Cpa4
Q8BU03	370330			484870	102910	Periodic tryptophan protein 2 homolog OS=Mus musculus
Q8K4L3				405810	243159	Supervillin OS=Mus musculus OX=10090
Q61024	119530	279070			64283	Asparagine synthetase [glutamine-hydrolyzing] OS=Mus musculus
Q9QXK7		211520	42924		77505	Cleavage and polyadenylation specificity factor subunit 3 OS=Mus musculus
Q5U4D9		554070	299550	563520	37315	THO complex subunit 6 homolog OS=Mus musculus OX=10090
Q9WTX2	654990			881580	34371	Interferon-inducible double-stranded RNA-dependent protein kinase activator A OS=Mus
Q8BJW6	1485900	1168000		793650	64403	Eukaryotic translation initiation factor 2A OS=Mus musculus
P17742		0			17971	Peptidyl-prolyl cis-trans isomerase A OS=Mus musculus OX=10090
V9GX31		0			8561	Peptidyl-prolyl cis-trans isomerase OS=Mus musculus OX=10090
Q70FJ1	87369	216740			436231	A-kinase anchor protein 9 OS=Mus musculus OX=10090 GN=Akap9
Q9CWX4		339410		544520	42407	Mitochondrial RNA pseudouridine synthase Rpusd4 OS=Mus
Q9CPR1		404110			21138	RWD domain-containing protein 4 OS=Mus musculus OX=10090
Q9CY66				242140	23474	H/ACA ribonucleoprotein complex subunit 1
Q9JJW6			388040		23730	Aly/REF export factor 2 OS=Mus musculus OX=10090 GN=Alyref2

O08583			388040		26940	THO complex subunit 4 OS=Mus musculus OX=10090 GN=Alyref
Q9CR26	239050	354900		310330	33913	Vacuolar protein sorting-associated protein VTA1 homolog OS=Mus musculus OX=10090
E9QAE1	9284500	8179900		4840400	209076	NCK-associated protein 5 OS=Mus musculus OX=10090 GN=Nckap5
Q923G2				214900	17143	DNA-directed RNA polymerases I II and III subunit RPABC3 OS=Mus musculus
Q8BTU1	1231400			1877300	22748	Cilia- and flagella-associated protein 20 OS=Mus musculus
P47758	0				29579	Signal recognition particle receptor subunit beta OS=Mus musculus
E9Q035	0				107796	Predicted gene 20425 OS=Mus musculus OX=10090
Q8VD00		1401500		865680	20811	Sigma intracellular receptor 2 OS=Mus musculus OX=10090
Q60520	82856	118240			145088	Paired amphipathic helix protein Sin3a OS=Mus musculus OX=10090
O54879	1094700	723620			23010	High mobility group protein B3 OS=Mus musculus OX=10090
Q921M4	140130	236910		0	113278	Golgin subfamily A member 2 OS=Mus musculus OX=10090
Q9JJ89				631960	46487	Coiled-coil domain-containing protein 86 OS=Mus musculus
O55143		556880		260250	114858	Sarcoplasmic/endoplasmic reticulum calcium ATPase 2 OS=Mus
Q5PR73	5311500	4837300	4467800	5175300	22498	GTP-binding protein Di-Ras2 OS=Mus musculus OX=10090 GN=Diras2
Q80U49	128000	0			170820	Centrosomal protein of 170 kDa protein B OS=Mus musculus
P51432		0			139487	1-phosphatidylinositol 4 5-bisphosphate phosphodiesterase beta-3 OS=Mus musculus
Q9JHI7	522560	256650	319270	459270	48937	Exosome complex component RRP45 OS=Mus musculus
O08756		639410	35355		27419	3-hydroxyacyl-CoA dehydrogenase type-2 OS=Mus musculus
D3Z0M9	158200				95495	DEAD (Asp-Glu-Ala-Asp) box polypeptide 23 OS=Mus musculus
Q8CAQ8	163310				83900	MICOS complex subunit Mic60 OS=Mus musculus OX=10090
O08709		136850			24871	Peroxiredoxin-6 OS=Mus musculus

Q9DC28	486270	951430		609290	47316	Casein kinase I isoform delta OS=Mus musculus OX=10090 GN=Csnk1d
Q9JMK2	486270	951430		609290	47322	Casein kinase I isoform epsilon OS=Mus musculus OX=10090
Q8BTW3		314530			28370	Exosome complex component MTR3 OS=Mus musculus
Q91WQ3	398350	363180		0	59105	Tyrosine--tRNA ligase cytoplasmic OS=Mus musculus OX=10090
Q99LC8	519280			415020	33816	Translation initiation factor eIF-2B subunit alpha OS=Mus musculus
Q99LH2	356320			287660	55604	Phosphatidylserine synthase 1 OS=Mus musculus OX=10090
A2BGJ5	506750	505540		736020	41346	Zinc finger MYND domain-containing 12 OS=Mus musculus
Q8R5K4	686330	1014200		1017000	129228	Nucleolar protein 6 OS=Mus musculus
Q8BG32		280040			47437	26S proteasome non-ATPase regulatory subunit 11 OS=Mus
Q8C3X8	0	161220	0		79997	Lipase maturation factor 2 OS=Mus musculus OX=10090 GN=Lmf2
Q9DBG6		2036000			69063	Dolichyl-diphosphooligosaccharide--protein glycosyltransferase
Q8R010	146060	489510			35378	Aminoacyl tRNA synthase complex-interacting multifunctional protein 2
Q91V92		0			119728	ATP-citrate synthase OS=Mus musculus OX=10090 GN=Acly
Q9QXS6				166790	77287	Drebrin OS=Mus musculus OX=10090
Q9DC69		125660			42525	NADH dehydrogenase [ubiquinone] 1 alpha subcomplex subunit 9 mitochondrial OS=Mus
Q61187	181960	0	94504		44124	Tumor susceptibility gene 101 protein OS=Mus musculus
Q80XC2		384750			31639	tRNA (adenine(58)-N(1))-methyltransferase catalytic subunit TRMT61A OS=Mus
O88665			487420	906450	74000	Bromodomain-containing protein 7 OS=Mus musculus OX=10090
Q91YU8		511160			52756	Suppressor of SWI4 1 homolog OS=Mus musculus OX=10090
Q8VCC9		1091500	259960	297810	90821	Spondin-1 OS=Mus musculus OX=10090
Q9JLF6	0				89826	Protein-glutamine gamma-glutamyltransferase K OS=Mus musculus

Q9DCA5		409800			41241	Ribosome biogenesis protein BRX1 homolog OS=Mus musculus
P00405		0			25976	Cytochrome c oxidase subunit 2 OS=Mus musculus OX=10090
Q91X79		648660		529040	28901	Chymotrypsin-like elastase family member 1 OS=Mus musculus
P47856				204420	78539	Glutamine--fructose-6-phosphate aminotransferase [isomerizing] 1 OS=Mus
P12367		0			45389	cAMP-dependent protein kinase type II-alpha regulatory subunit OS=Mus musculus
E9Q7E2				0	195987	AT-rich interactive domain-containing protein 2 OS=Mus
Q9CRA8				1059100	25194	Exosome complex component RRP46 OS=Mus musculus
Q8K202			280140		54034	DNA-directed RNA polymerase I subunit RPA49 OS=Mus
Q791V5		80491			33499	Mitochondrial carrier homolog 2 OS=Mus musculus OX=10090
Q5XG71	0				317744	Small subunit processome component 20 homolog OS=Mus
P62245	321070	130560	356870		14840	40S ribosomal protein S15a OS=Mus musculus OX=10090 GN=Rps15a
Q8R3L2	970480			758590	76685	Transcription factor 25 OS=Mus musculus OX=10090 GN=Tcf25
Q8BW10		0			45464	RNA-binding protein NOB1 OS=Mus musculus OX=10090
G3XA57	241530				58207	Rab11 family-interacting protein 2 OS=Mus musculus OX=10090
Q9D620	241530				70684	Rab11 family-interacting protein 1 OS=Mus musculus OX=10090
Q8CHP5				348570	22690	Partner of Y14 and mago OS=Mus musculus OX=10090 GN=Pym1
Q9D6X6	647850		331210		43072	Serine protease 23 OS=Mus musculus
Q61171		256330			21779	Peroxiredoxin-2 OS=Mus musculus
P61965		1101900	200210		36589	WD repeat-containing protein 5 OS=Mus musculus OX=10090
E9Q6A2		2007600			44193	Serpin peptidase inhibitor clade E (nexin plasminogen activator inhibitor type 1) member
Q922Q4		123400		98036	33659	Pyrroline-5-carboxylate reductase 2 OS=Mus musculus OX=10090

Q9CZI9	353880				37309	Apoptosis-enhancing nuclease OS=Mus musculus OX=10090
P80316		534140		359310	59624	T-complex protein 1 subunit epsilon OS=Mus musculus OX=10090
Q792Y8	464860				26119	MCG15081 OS=Mus musculus OX=10090 GN=Gm10334 PE=3
Q792Z0	464860				26184	Protease serine 3 OS=Mus musculus
Q07417	333470	484790	221200		44890	Short-chain specific acyl-CoA dehydrogenase mitochondrial OS=Mus musculus OX=10090
O88543	106360	134850		136870	47832	COP9 signalosome complex subunit 3 OS=Mus musculus
P62334	380430	848020			44173	26S proteasome regulatory subunit 10B OS=Mus musculus
Q9ER67	0	0			65401	Maged2 protein OS=Mus musculus OX=10090
Q7TT50				0	194751	Serine/threonine-protein kinase MRCK beta OS=Mus musculus
Q61191		446610	121130	197470	210435	Host cell factor 1 OS=Mus musculus
Q8BHF7		103360		0	62489	CDP-diacylglycerol--glycerol-3-phosphate 3-phosphatidyltransferase mitochondrial OS=Mus
Q9D0D3		0			65229	Poly(A) RNA polymerase mitochondrial OS=Mus musculus OX=10090
Q8K4G5	337500	420180		512010	96805	Actin-binding LIM protein 1 OS=Mus musculus OX=10090
P28301	1022100	730510	391370	439840	46701	Protein-lysine 6-oxidase OS=Mus musculus OX=10090 GN=Lox
Q80UZ2	0				79559	Protein SDA1 homolog OS=Mus musculus OX=10090 GN=Sdad1
Q8BTH8	111480	217700		171260	52744	Casein kinase I isoform gamma-1 OS=Mus musculus OX=10090
Q7TT37		125510			149583	Elongator complex protein 1 OS=Mus musculus OX=10090
O55222	691710	417780			51373	Integrin-linked protein kinase OS=Mus musculus OX=10090
Q8C181		0			40156	Muscleblind-like protein 2 OS=Mus musculus OX=10090 GN=Mbnl2
A2AGT5	150980				225633	Cytoskeleton-associated protein 5 OS=Mus musculus OX=10090
Q6PB66		83474			156614	Leucine-rich PPR motif-containing protein mitochondrial OS=Mus musculus OX=10090

P97857	92850				105801	A disintegrin and metalloproteinase with thrombospondin motifs 1 OS=Mus musculus
B9EJ86	60981				101269	Oxysterol-binding protein-related protein 8 OS=Mus musculus
Q8CHY6	242010				67334	Transcriptional repressor p66 alpha OS=Mus musculus OX=10090
O88986				975420	44931	2-amino-3-ketobutyrate coenzyme A ligase mitochondrial OS=Mus musculus OX=10090
Q62181				126320	85289	Semaphorin-3C OS=Mus musculus OX=10090
P49135			54516		89127	General transcription and DNA repair factor IIH helicase subunit XPB OS=Mus musculus
Q9DCN2		221920			34128	NADH-cytochrome b5 reductase 3 OS=Mus musculus OX=10090
Q9D4J7		164040			41139	PHD finger protein 6 OS=Mus musculus OX=10090 GN=Phf6
Q6ZQ38		111900		191210	136331	Cullin-associated NEDD8-dissociated protein 1 OS=Mus
Q8K205			235330		117367	Processing of 1 ribonuclease P/MRP family (S. cerevisiae) OS=Mus musculus
A6H687	692690		663280		46384	SAC3 domain-containing protein 1 OS=Mus musculus OX=10090
P19221		271530			70269	Prothrombin OS=Mus musculus OX=10090
P63001	738050	481430			21450	Ras-related C3 botulinum toxin substrate 1 OS=Mus musculus
P60764	412590	481430			21379	Ras-related C3 botulinum toxin substrate 3 OS=Mus musculus
Q05144	412590	481430			21441	Ras-related C3 botulinum toxin substrate 2 OS=Mus musculus
Q9Z1X4		304300			96021	Interleukin enhancer-binding factor 3 OS=Mus musculus OX=10090
P55096		380380		344730	75475	ATP-binding cassette sub-family D member 3 OS=Mus musculus
P19182		294560			49935	Interferon-related developmental regulator 1 OS=Mus musculus
Q9DBS5	1278000	1379100	1190800		68613	Kinesin light chain 4 OS=Mus musculus OX=10090 GN=Klc4
Q9WVK4	472500			438500	60603	EH domain-containing protein 1 OS=Mus musculus OX=10090
Q8BH64	472500			438500	61175	EH domain-containing protein 2 OS=Mus musculus OX=10090

P52432			122590		39107	DNA-directed RNA polymerases I and III subunit RPAC1 OS=Mus
P51174	411550	493050			47908	Long-chain specific acyl-CoA dehydrogenase mitochondrial OS=Mus musculus OX=10090
Q91YN9		0			23474	BAG family molecular chaperone regulator 2 OS=Mus musculus
Q9D7N3	375140	472430	157690		44929	28S ribosomal protein S9 mitochondrial OS=Mus musculus OX=10090
Q9QZQ1				259090	206497	Afadin OS=Mus musculus OX=10090
Q61598		318170			50537	Rab GDP dissociation inhibitor beta OS=Mus musculus OX=10090
Q9QYI8				0	35626	DnaJ homolog subfamily B member 7 OS=Mus musculus OX=10090
O54946				0	39807	DnaJ homolog subfamily B member 6 OS=Mus musculus OX=10090
Q9D2G2	644070		357390	0	48995	Dihydrolipoyllysine-residue succinyltransferase component of 2-oxoglutarate
G5E8A8			0		62735	Tektin-5 OS=Mus musculus OX=10090
Q80X66	1009500	0	2089200	487910	53772	BTB/POZ domain-containing protein 10 OS=Mus musculus
Q8C460	634720	771650	464650		37189	ERI1 exoribonuclease 3 OS=Mus musculus OX=10090 GN=Eri3
Q6P9K8	571570	675540		687450	150495	Caskin-1 OS=Mus musculus OX=10090
Q921U8		584700		582030	100289	Smoothelin OS=Mus musculus OX=10090
Q69ZL1	1952500	2799800		0	155169	FYVE RhoGEF and PH domain-containing protein 6 OS=Mus
Q9D903			295620		34703	Probable rRNA-processing protein EBP2 OS=Mus musculus
Q91V12		126780			42537	Cytosolic acyl coenzyme A thioester hydrolase OS=Mus musculus
Q8CBW3		290510			52288	Abl interactor 1 OS=Mus musculus OX=10090
Q02053	347130	960680			117809	Ubiquitin-like modifier-activating enzyme 1 OS=Mus musculus
H3BK29			1289300		102849	Vomeronasal 2 receptor 6 OS=Mus musculus OX=10090 GN=Vmn2r6
Q8JZS9	489580		401430	581070	23953	39S ribosomal protein L48 mitochondrial OS=Mus musculus
A2A863	257970			0	201649	Integrin beta-4 OS=Mus musculus OX=10090
E9Q4G9		409850			30157	Predicted gene 21293 OS=Mus musculus OX=10090

L7N2C4		409850			30362	Predicted gene 4340 OS=Mus musculus OX=10090 GN=Gm4340
E9PZY4		409850			32988	Predicted gene 4303 OS=Mus musculus OX=10090 GN=Gm4307
E9PX25		409850			33628	Predicted gene 4302 OS=Mus musculus OX=10090 GN=Gm4302
O35129		397040			33296	Prohibitin-2 OS=Mus musculus OX=10090
P10417			90535		26407	Apoptosis regulator Bcl-2 OS=Mus musculus OX=10090 GN=Bcl2
Q61136	114320			0	116976	Serine/threonine-protein kinase PRP4 homolog OS=Mus musculus
A0A0B4J1H6	451950	1118800			13131	Immunoglobulin kappa chain variable 2-137 (Fragment) OS=Mus musculus OX=10090
P01630	451950	1118800			12496	Ig kappa chain V-II region 7S34.1 OS=Mus musculus OX=10090
P52293	282700				57928	Importin subunit alpha-1 OS=Mus musculus OX=10090 GN=Kpna2
Q5F259			244540		70374	Ankyrin repeat domain-containing protein 13B OS=Mus musculus
Q8BSQ9				119280	187187	Protein polybromo-1 OS=Mus musculus OX=10090 GN=Pbrm1
E9PVA8			48756		293018	eIF-2-alpha kinase activator GCN1 OS=Mus musculus OX=10090
Q8C547				21887	224318	HEAT repeat-containing protein 5B OS=Mus musculus OX=10090
P63085				61572	41276	Mitogen-activated protein kinase 1 OS=Mus musculus OX=10090
Q63844				61572	43067	Mitogen-activated protein kinase 3 OS=Mus musculus OX=10090
Q00342				61572	113496	Receptor-type tyrosine-protein kinase FLT3 OS=Mus musculus
P26618				61572	122683	Platelet-derived growth factor receptor alpha OS=Mus musculus
P05622				61572	122790	Platelet-derived growth factor receptor beta OS=Mus musculus
P35917				61572	153016	Vascular endothelial growth factor receptor 3 OS=Mus musculus
O54949				61572	58313	Serine/threonine-protein kinase NLK OS=Mus musculus OX=10090
Q80Y86				61572	60679	Mitogen-activated protein kinase 15 OS=Mus musculus
P35969				61572	149876	Vascular endothelial growth factor receptor 1 OS=Mus musculus

P35918				61572	152516	Vascular endothelial growth factor receptor 2 OS=Mus musculus
A2AAJ9	0				966606	Obscurin OS=Mus musculus OX=10090
Q9D1Q5	664990	414810			44558	MCG21235 OS=Mus musculus OX=10090 GN=Serpinb3b PE=2
Q6UKZ0	664990	414810			44771	MCG8992 OS=Mus musculus OX=10090 GN=Serpinb3d PE=2
A2RSF9	664990	414810			44370	Serine (Or cysteine) peptidase inhibitor clade B member 3C OS=Mus musculus OX=10090
G3X9V8	664990	414810			44453	MCG129038 OS=Mus musculus OX=10090
Q9WVB4		387280	578500	1538800	167726	Slit homolog 3 protein OS=Mus musculus OX=10090 GN=Slit3
Q3UM18			1469700		73157	Large subunit GTPase 1 homolog OS=Mus musculus OX=10090
Q922J9	0			0	59435	Fatty acyl-CoA reductase 1 OS=Mus musculus OX=10090 GN=Far1
Q9DC50	0		209130	0	70264	Peroxisomal carnitine O-octanoyltransferase OS=Mus musculus
Q91YJ3	260750	0	129300	277700	26178	Thymocyte nuclear protein 1 OS=Mus musculus OX=10090
Q91WM3				0	52107	U3 small nucleolar RNA-interacting protein 2 OS=Mus musculus
P61620	543110				52265	Protein transport protein Sec61 subunit alpha isoform 1 OS=Mus
Q9JLR1	543110				52248	Protein transport protein Sec61 subunit alpha isoform 2 OS=Mus
P80317	379840	2159900			58004	T-complex protein 1 subunit zeta OS=Mus musculus OX=10090
Q9DCN1				99971	51511	Peroxisomal NADH pyrophosphatase NUDT12 OS=Mus
Q80VI1		56354			79513	E3 ubiquitin-protein ligase TRIM56 OS=Mus musculus OX=10090
Q8CJF7	825760				247644	Protein ELYS OS=Mus musculus OX=10090
Q3UB74		579290			44881	Transforming growth factor beta regulator 1 OS=Mus musculus
O35593		215980		0	34577	26S proteasome non-ATPase regulatory subunit 14 OS=Mus
Q8CD15			94893		53517	Ribosomal oxygenase 2 OS=Mus musculus OX=10090 GN=Riox2
A0A1B0GR13		0			71774	Cylicin basic protein of sperm head cytoskeleton 1 OS=Mus musculus

Q925B0	0				35908	PRKC apoptosis WT1 regulator protein OS=Mus musculus
Q9EST1	278830				49593	Gasdermin-A OS=Mus musculus OX=10090
P27773	86330				56678	Protein disulfide-isomerase A3 OS=Mus musculus OX=10090
Q3UXZ9	17532				192215	Lysine-specific demethylase 5A OS=Mus musculus OX=10090
Q9D051		0			38937	Pyruvate dehydrogenase E1 component subunit beta mitochondrial OS=Mus musculus
Q8C147		0			238976	Dedicator of cytokinesis protein 8 OS=Mus musculus OX=10090
Q8R1A4		0			241436	Dedicator of cytokinesis protein 7 OS=Mus musculus OX=10090
P15105	1310500	1115700		884710	42120	Glutamine synthetase OS=Mus musculus OX=10090 GN=Glul
Q640M1		323530			87265	U3 small nucleolar RNA-associated protein 14 homolog A OS=Mus musculus OX=10090
Q9JLZ6		439240			66767	Hypermethylated in cancer 2 protein OS=Mus musculus OX=10090
A0A0N4SVP8				315030	46959	Predicted pseudogene 5580 OS=Mus musculus OX=10090 GN=Gm5580
E9PV04				315030	46854	Predicted gene 8994 OS=Mus musculus OX=10090 GN=Gm8994
Q91VC3				315030	46840	Eukaryotic initiation factor 4A-III OS=Mus musculus OX=10090
Q8BIL5		95067			84439	Protein Hook homolog 1 OS=Mus musculus OX=10090 GN=Hook1
Q91X78				77951	39182	Erlin-1 OS=Mus musculus OX=10090
Q8BFZ9				77951	37873	Erlin-2 OS=Mus musculus OX=10090
Q9CWG8				0	48385	Protein arginine methyltransferase NDUFAF7 mitochondrial OS=Mus
Q8VCH0		123040			43995	3-ketoacyl-CoA thiolase B peroxisomal OS=Mus musculus OX=10090
Q921H8		123040			43953	3-ketoacyl-CoA thiolase A peroxisomal OS=Mus musculus OX=10090
P70419		0			72932	Polypeptide N-acetylgalactosaminyltransferase 3 OS=Mus musculus OX=10090
Q9Z2C4	2546200	2826600	2954700		75314	Myotubularin-related protein 1 OS=Mus musculus OX=10090
Q8VC77				8456300	36857	Akr1c20 protein OS=Mus musculus

P23249		99739			113583	Putative helicase MOV-10 OS=Mus musculus OX=10090 GN=Mov10
P42208		1409600		851370	41526	Septin-2 OS=Mus musculus OX=10090
E9PVZ8		3722000	0	0	370154	Golgi autoantigen golgin subfamily b macrogolgin 1 OS=Mus musculus
O88207	0		172050		183676	Collagen alpha-1(V) chain OS=Mus musculus OX=10090 GN=Col5a1
E9Q7L1			256280		171618	URB2 ribosome biogenesis 2 homolog (S. cerevisiae) OS=Mus
Q8BXR5				0	200440	Sodium leak channel non-selective protein OS=Mus musculus
Q6NZQ2		65692			76913	Probable ATP-dependent RNA helicase DDX31 OS=Mus musculus
Q64518			174380		113638	Sarcoplasmic/endoplasmic reticulum calcium ATPase 3 OS=Mus
Q7M6Z4				0	158955	Kinesin-like protein KIF27 OS=Mus musculus OX=10090
Q6ZQ58			291210		121125	La-related protein 1 OS=Mus musculus
P18242	103430		157040		44954	Cathepsin D OS=Mus musculus OX=10090
Q80WR5		0			24774	UPF0688 protein C1orf174 homolog OS=Mus musculus
Q61176	378090				34808	Arginase-1 OS=Mus musculus OX=10090
Q5DTX6	0	0			144797	Junctional protein associated with coronary artery disease OS=Mus musculus OX=10090
Q8BTV1	769100	882410			39548	Tumor suppressor candidate 3 OS=Mus musculus OX=10090
Q8K0J2				0	45379	UDP-GlcNAc:betaGal beta-1 3-N-acetylglucosaminyltransferase 7 OS=Mus
Q8C052	0	148910			102939	Microtubule-associated protein 1S OS=Mus musculus OX=10090
Q91VS8		210210			121280	FERM ARHGEF and pleckstrin domain-containing protein 2 OS=Mus musculus
Q9D0I8	674610				27546	mRNA turnover protein 4 homolog OS=Mus musculus OX=10090
Q8K1A6				127400	103698	Coiled-coil and C2 domain-containing protein 1A OS=Mus
Q8C5N3				1619500	104774	Pre-mRNA-splicing factor CWC22 homolog OS=Mus musculus
A2AK42				1619500	95672	Predicted gene 13691 OS=Mus musculus OX=10090

Q8K3A9		153030			72050	7SK snRNA methylphosphate capping enzyme OS=Mus
Q9R1V4	0				84134	Disintegrin and metalloproteinase domain-containing protein 11 OS=Mus
E9Q4F7	0				296184	Ankyrin repeat domain-containing protein 11 OS=Mus musculus
Q99M74	539080				57140	Keratin type II cuticular Hb2 OS=Mus musculus OX=10090 GN=Krt82
D3Z2X2	937320		570970		536540	Dynein heavy chain domain 1 OS=Mus musculus OX=10090
Q9ERL9		97651			77588	Guanylate cyclase soluble subunit alpha-1 OS=Mus musculus
P70662		133450000	89534000		46503	LIM domain-binding protein 1 OS=Mus musculus OX=10090
Q9JKV1		179310			42060	Proteasomal ubiquitin receptor ADRM1 OS=Mus musculus
Q8BJY1		0			55972	26S proteasome non-ATPase regulatory subunit 5 OS=Mus
Q91ZU6	470420	386050			834234	Dystonin OS=Mus musculus OX=10090
Q9WU78		108670			96024	Programmed cell death 6-interacting protein OS=Mus musculus
Q9WTP9			0		31702	Ventral anterior homeobox 2 OS=Mus musculus OX=10090
Q8CI51		512780			63299	PDZ and LIM domain protein 5 OS=Mus musculus OX=10090
B1ART2			4679200		490973	Vacuolar protein sorting 13D OS=Mus musculus OX=10090 GN=Vps13d
Q9D483				5557700	60706	DNA-directed RNA polymerase III subunit RPC3 OS=Mus musculus
Q9ES52		396680			133542	Phosphatidylinositol 3 4 5-trisphosphate 5-phosphatase 1 OS=Mus musculus OX=10090
